# Investigating the Proton Donor in the NO Reductase from *Paracoccus denitrificans*

**DOI:** 10.1371/journal.pone.0152745

**Published:** 2016-03-31

**Authors:** Josy ter Beek, Nils Krause, Pia Ädelroth

**Affiliations:** Department of Biochemistry and Biophysics, Stockholm University, Stockholm, Sweden; Universidade Nova de Lisboa, PORTUGAL

## Abstract

Variant nomenclature: the variants were made in the NorB subunit if not indicated by the superscript ^c^, which are variants in the NorC subunit (e.g. E122A = exchange of Glu-122 in NorB for an Ala, E71^c^D; exchange of Glu-71 in NorC for an Asp).

Bacterial NO reductases (NORs) are integral membrane proteins from the heme-copper oxidase superfamily. Most heme-copper oxidases are proton-pumping enzymes that reduce O_2_ as the last step in the respiratory chain. With electrons from cytochrome *c*, NO reductase (*c*NOR) from *Paracoccus (P*.*) denitrificans* reduces NO to N_2_O via the following reaction: 2NO+2e^-^+2H^+^→N_2_O+H_2_O. Although this reaction is as exergonic as O_2_-reduction, *c*NOR does not contribute to the electrochemical gradient over the membrane. This means that *c*NOR does not pump protons and that the protons needed for the reaction are taken from the periplasmic side of the membrane (since the electrons are donated from this side). We previously showed that the *P*. *denitrificans c*NOR uses a single defined proton pathway with residues Glu-58 and Lys-54 from the NorC subunit at the entrance. Here we further strengthened the evidence in support of this pathway. Our further aim was to define the continuation of the pathway and the immediate proton donor for the active site. To this end, we investigated the region around the calcium-binding site and both propionates of heme *b*_3_ by site directed mutagenesis. Changing single amino acids in these areas often had severe effects on *c*NOR function, with many variants having a perturbed active site, making detailed analysis of proton transfer properties difficult. Our data does however indicate that the calcium ligation sphere and the region around the heme *b*_3_ propionates are important for proton transfer and presumably contain the proton donor. The possible evolutionary link between the area for the immediate donor in *c*NOR and the proton loading site (PLS) for pumped protons in oxygen-reducing heme-copper oxidases is discussed.

## Introduction

NO reductases (NORs) convert NO to N_2_O and H_2_O (2NO + 2H^+^ + 2e^-^ → N_2_O + H_2_O). They are found in denitrifying bacteria, but also in pathogens that use them to evade the toxic NO that is produced by the host during the immune response. Cytochrome *c* dependent NORs (*c*NORs) are integral membrane proteins and members of the heme copper oxidase (HCuO) superfamily. Most HCuO family members reduce oxygen to water, and use the available energy from oxygen reduction to contribute to the electrochemical proton gradient over the membrane. This is done by taking the electrons from donors on the (positive, lower pH) outside, and the protons from the (negative, higher pH) inside, and by an additional reaction where protons are actively translocated (pumped) over the membrane. *c*NORs, such as the one from *P*. *denitrificans*, on the other hand, have been shown not to contribute to the electrochemical potential, even though the energy from NO reduction is as large as from oxygen reduction [1,2]. *c*NORs are also capable of oxygen reduction, but also then no electrochemical potential is formed. Since electrons are donated by cytochrome (cyt.) *c* (or pseudoazurin, see Ref. [3]) from the outside, protons also have to be delivered from this side, such that there must be a proton transfer pathway leading from the periplasmic bulk solution into the active site in *c*NOR.

**Fig 1:**
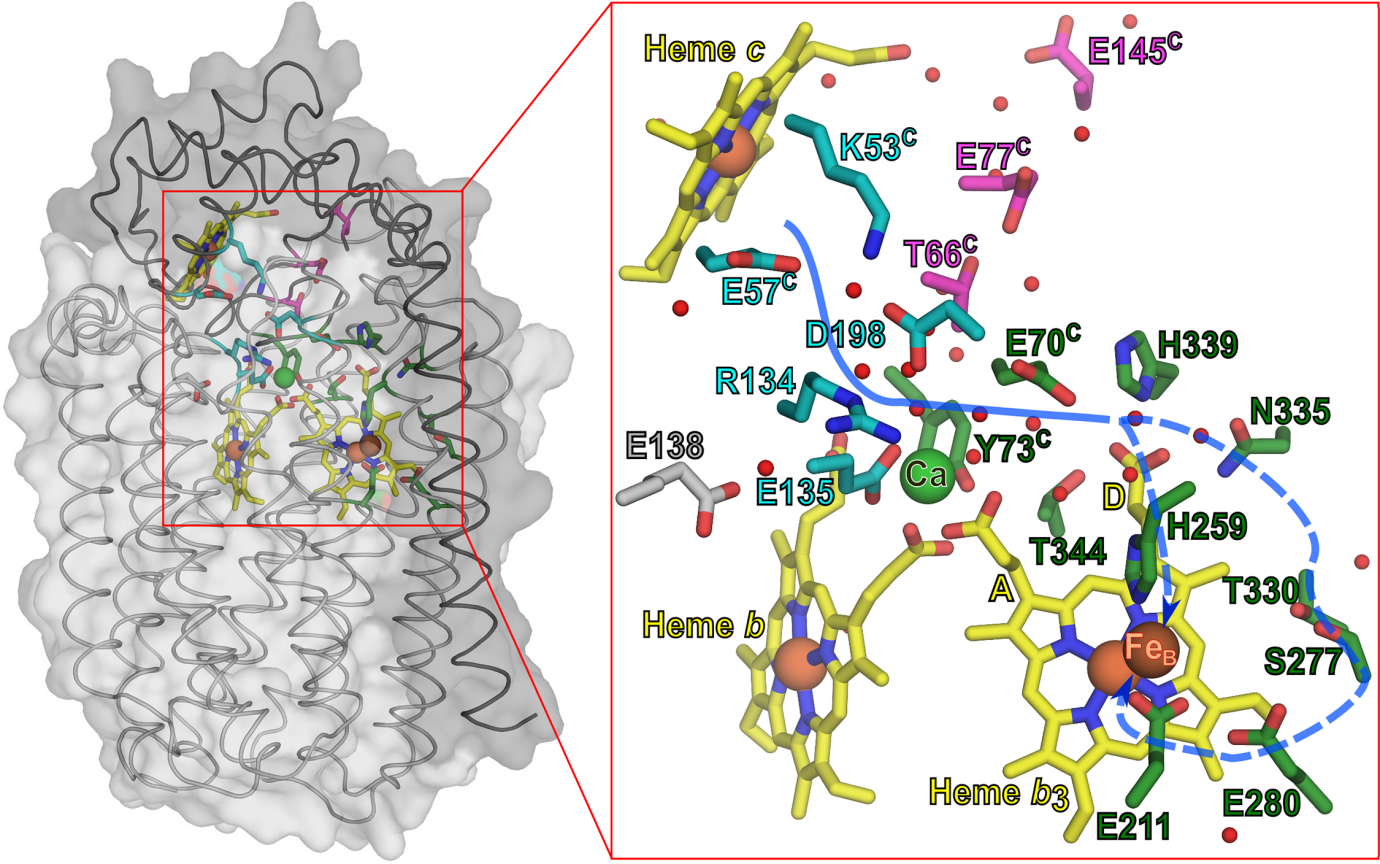
*c*NOR structure with the suggested proton pathway and the investigated residues. Structure of *c*NOR from *Ps*. *aeruginosa* (Proton Data Bank code 3O0R [5]). The NorB (light grey) and NorC (dark grey) subunits are shown in surface and cartoon representation on the left. Hemes and side-chains of the discussed residues are shown in stick representation. All Fe^3+^ and Ca^2+^ ions are shown as spheres. The residues of proton pathway 1 are shown in cyan, the investigated residues of pathway 2 are shown in pink. The residues that are predicted to lead the proton to the active site are shown in dark green. Small red spheres indicate crystallographic waters within 3.5 Å of the shown residues, heme propionates and metal sites. The A and D-propionate of the *b*_*3*_ heme are also indicated. Blue (dotted) lines indicate the suggested proton pathway.

*c*NOR is purified as a heterodimer of the subunits NorB and NorC (see Fig 1). NorB consists of 12 transmembrane helices and contains two *b*-hemes. The propionates of these hemes are bridged via a calcium ion. The *b*_3_-heme and a non-heme iron (Fe_B_) form the binuclear center, which binds and reduces NO. The additional subunit NorC (residues from this subunit are indicated with a c in superscript), has a single transmembrane helix and a large periplasmic domain with a cytochrome *c* fold. The *c*-heme in this domain is the presumed site of electron entry.

Proton transfer pathways in proteins generally consist of hydrogen-bonded chains of water molecules (stabilised by polar amino acids) and/or protonatable amino acids (see Ref. [4] for a review). Based on the crystal structure [5] and molecular dynamic simulations [6] of the *c*NOR from *Pseudomonas (Ps*.*) aeruginosa*, three such putative proton pathways (termed pathway 1–3) were predicted. In a recent study using the *P*. *denitrificans c*NOR (53% and 49% sequence identity with the NorB and NorC subunits from *Ps*. *aeruginosa*, respectively), we presented evidence that only a single proton pathway was used in *c*NOR [7], namely pathway 1 with residues Glu^c^ -58 and Lys^c^ -54 (from the NorC subunit) at the entrance (E57^c^ and K53^c^ in *Ps*. *aeruginosa*, see Fig 1).

We have previously investigated proton transfer in *c*NOR by studying effects on the proton-coupled electron transfer reaction (called ETPT) during the single-turnover reaction between fully reduced *c*NOR and oxygen [2,7–10]. We also showed that the reaction with O_2_ is a good model for the physiological reaction with NO in terms of proton transfer characteristics e.g. showing similar rate constants [2] (see also S1 Fig), and good correlations between effects by e.g. mutation on turnover rates with both substrates [7,8,11,12]. Interpreting data from the reaction between *c*NOR and NO is complicated by the substrate inhibition observed at higher [NO] [10] and because fully reduced *c*NOR can do two full turnovers, reducing four NO molecules. The reaction with O_2_ has neither of these two complications. Moreover, the rate constant for the ETPT during O_2_ reduction is rate-limited by proton transfer (shown by a substantial solvent isotope effect [7]) and depends on pH in a way that is well fitted to a model with an internal proton donor (in rapid equilibrium with the bulk solution), which delivers protons to the active site [7,8]. This proton donor has a p*K*a of 6.6 and is a protein-internal group, possibly a protonatable amino acid, a water molecule, or a heme propionate (as discussed in [7]). Since the ETPT reaction is rate limited by proton transfer, it is sensitive to changes in proton-transfer properties. We therefore study the ETPT reaction in variants with residues putatively involved in proton transfer exchanged, to explore the roles played by these residues in the proton transfer dynamics.

Previously a large shift in the p*K*a of the proton donor was found when E122 (E135 in *Ps*. *aeruginosa*, Fig 1) was substituted [9]. Exchanging E125 (E138 in *Ps*. *aeruginosa*), located nearby in the same loop, led to a complete loss of activity. The large effects indicated that the proton donor is in this region [9]. However, detailed analysis was hampered by the lack of detailed structural information. The crystal structure later showed that these residues are buried inside the membrane protein and identified the E135 as a Ca^2+^ ligand (*Ps*. *aeruginosa* numbering: the Ca^2+^ is further coordinated by Gly^C^-71 (backbone C = O), Tyr^C^-73, and one of the propionates from both hemes *b* and *b*_3_ see Fig 1) [5]. It is not clear if these glutamates play a direct role in proton transfer or if it is the change in Ca^2+^ ligation that in turn alters the proton transfer properties.

In contrast to our results with the *P*. *denitrificans c*NOR, Schurig-Briccio et al. [13] concluded in a mutagenesis study of the *c*NOR from *Thermus thermophilus* (38% identity with the NorB subunits and 28% with the NorC subunits of both *Ps*. *aeruginosa* and *P*. *denitrificans*), that no universally shared specific pathway was used in all *c*NORs. To further study how specifically proton transfer pathway 1 is used in the *P*. *denitrificans c*NOR, new variants were constructed by exchanging conserved residues of the proposed pathways. With the additional aim of identifying the p*K*a = 6.6 proton donor, residues near the calcium and the heme *b*_3_ propionates were exchanged. Because of the evolutionary relationship between NORs and the O_2_-reducing HCuOs, the *c*NOR proton input path might be evolutionary related to the proton-pumping path leading from the active site outwards for O_2_-reducing HCuOs. Thus, investigating the proton donor in *c*NOR could also shed light on the location of the so far un-identified proton-loading site (PLS) for the pumped protons in the pumping heme copper oxidases.

Data from this study further strengthens the evidence that only pathway 1 is used for proton transfer in *c*NOR from *P*. *denitrificans*. It proved more difficult to determine the continuation of the proton transfer pathway or to specifically identify the proton donor, since mutating residues near the Ca^2+^ and the heme propionates leads to multiple effects on *c*NOR properties, which cannot unequivocally be assigned to effects on proton transfer. However, taking results from all data together, the suggested location of the proton donor in the region of the heme *b*_3_ propionates/Ca^2+^ site is further strengthened. This area overlaps with that suggested to contain the loading site for pumped protons in the proton-pumping HCuOs.

## Materials and Methods

### Cloning, Expression, and Purification

For *c*NOR expression the pNOREX plasmid was used [11]. Mutations were introduced by using the QuickChange XL site-directed mutagenesis kit (Stratagene). The pNOREX plasmids were transformed to the *E*. *coli* JM109 strain containing the pEC86 vector as previously described [11]. Freshly transformed *E*. *coli* was grown and *c*NOR was expressed as described in Ref. [9].

Minor changes were made to the purification procedure in Ref. [7], our current procedure is shortly described as followed. For *c*NOR purification, membrane vesicles from a 6 liter cell culture (around 20 mL of membranes; ~20 g with ~10 g of proteins) were solubilized in 100 mL buffer containing 100 mM Tris, pH 7.6, 50 mM NaCl, 1 mM EDTA, and 1% (w/v) n-dodecyl-β-D-maltoside (DDM). The solution was incubated at 4°C with constant stirring for 1 h. Unsolubilized material was removed by centrifugation (30 min, 185,000 × g, 4°C), and the supernatant was filtered over a 0.2 μm filter. The filtrate was loaded at 5 mL/min on a 75 mL Q-Sepharose high performance (GE Healthcare) column pre-equilibrated with the same buffer and 0.04% (w/v) DDM. The column was washed with ~200 mL of 20 mM Tris, 250 mM NaCl, and 0.04% (w/v) DDM at 5 mL/min. *c*NOR was eluted from the column in a 472 mL gradient from 250 to 500 mM NaCl with 20 mM Tris, pH 7.6, and 0.04% (w/v) DDM at 4 mL/min. 4 mL fractions were collected and diluted 3× in 20 mM Tris, pH 7.6, and 0.04% (w/v) DDM. The absorbance spectra of the collected fractions were analyzed via a dip probe connected to a Cary 50 Bio spectrophotometer (Varian). Fractions with an A280 nm/A410 nm of < 2 were collected and concentrated over a 100 kDa cut-off filter (Millipore). The pooled fractions were diluted and reconcentrated in 20 mM Tris, pH 7.6, and 0.04% (w/v) DDM until the NaCl concentration was below 50 mM. 50 μl fractions of 50 to 650 μM purified protein (A280 nm/A410 nm ~1) were flash frozen in liquid nitrogen and stored at -80°C.

The presence of correctly inserted *b* and *c* hemes in all the *c*NOR variants was verified via UV-visible spectra from 260 to 700 nm on a Cary 50, 100 or 4000 spectrophotometer (Varian). The *c*NOR concentration was calculated from ε550 nm red-ox = 70 mM-1cm-1 [14].

### Kinetic measurements: multiple turnover, flash photolysis and flow flash

The multiple turnover reduction rates of the *c*NOR variants with NO were determined as described before [7]. Because of the substrate inhibition observed at high [NO], the given turnover rates are the maximal observed rates at ~5 μM NO.

Flash photolysis and flow-flash measurements were made as described previously [7]. Briefly, samples of ~5 μM *c*NOR were prepared in a modified Thunberg cuvette and measurements were made on a set-up described in Ref. [15] at T = 295K. The samples were made anaerobic on a vacuum-line and reduced with ascorbate. The reduced samples were put under 100% (v/v) CO (g) and incubated overnight at 4°C. CO recombination was measured by flash photolysis with the kinetic traces recorded at the indicated wavelength on a digital oscilloscope. CO was dissociated by a short laser flash at time zero. The conditions for flash photolysis were: ~5 μM *c*NOR in 50 mM HEPES pH 7.5, 50 mM KCl, and 0.05% (w/v) DDM, [CO] = 1 mM, T = 295K.

The reaction between fully reduced, CO-bound *c*NOR and O_2_ was studied using the flow-flash technique. The CO concentration was then lowered to ~30% (v/v, 70% N2 (g)) to slow the CO rebinding enough not to interfere with O_2_ binding. The protein sample was connected anaerobically to a stopped-flow syringe. Around 20 μM sodium dithionite was added to ensure complete anaerobicity during loading of the sample. The other syringe contained an oxygenated buffer. In the pH dependence measurements, different buffers at various pH values were used. The protein and the buffer samples were mixed in a 1:5 ratio in a modified stopped-flow apparatus (Applied Photophysics), and after a delay of 200 ms, a laser flash was applied to dissociate CO and allow O2 to bind and initiate the reaction. The conditions in flow-flash (after mixing) were: ~1 μM *c*NOR in 50 mM buffer, 50 mM KCl, 0.05% (w/v) DDM, [O2] = 1 mM, T = 295K. The type of buffer was dependent on the pH: MES (pH 6.0–7.0, HEPES (pH 7–8.5), Tris (pH 8.5), and citric acid (pH 6).

The absorbance changes were measured at 430 nm (mainly associated with changes at heme *b*_3_; used to measure CO or O_2_ dissociation/binding and follow the ETPT), 420 nm (redox changes of all three hemes present in *c*NOR contribute at this wavelength; used to measure CO dissociation/binding and follow the ETPT) and 550 nm (reporting specifically on the heme *c*, only the ETPT is visible).

### Data Handling and Analysis

The time course of the reaction was studied from microseconds to seconds at different wavelengths in the Soret and α regions as described previously [7]. For parts of the data a new custom-built set-up (Applied Photophysics) was used. Kinetic traces were recorded with a C9999 amplifier (Hamamatsu) on a digital oscilloscope. Only a single channel was used for recording without filtering and with 1 million points with 5% pre-trigger. The recording was over ~1 ms for flash photolysis, and over ~1 s for flow flash. The measured light-intensity changes were converted to absorbance and reduced to ~2,000 points by the Applied Photophysics software by averaging over a progressively increasing number of points.

For all shown or analysed data, 3 to 10 different traces were averaged. If needed, data was further smoothed in SigmaPlot (Systat). The time-resolved absorbance changes were fitted individually or globally to a model of consecutive irreversible reactions with the software package Pro-K (Applied Photophysics). The proton-coupled electron transfer phase was fitted based on averaged traces (at least 3 per wavelength) of 420 nm, 430 nm and 550 nm. The pH dependence of the proton-coupled electron transfer phase was fitted with the following equations [8]:

$$k_{\text{obs}}=\alpha_{\text{AH}}\left(\text{pH}\right)\cdot k_{H}$$ (Eq 1)

$$\alpha_{\text{AH}}\left(pH\right)=\frac{1}{1+10^{\text{pH}-{\text{p}K}_{\text{AH}}}}$$ (Eq 2)

where *k*obs represents the obtained rate constant at a certain pH, and *k*H is the maximum rate at low pH. *k*H is the rate-limiting internal proton transfer (in Ref. [7,8] presumed to be from a group, AH, assumed to be in rapid equilibrium with bulk pH, to the active site). αAH is the fraction of protonated AH, determined by its p*K*a and the pH. For wildtype as well as most *c*NOR variants (see Results), a small background rate, *k*0, was added to Eq 1 [7,8], making the maximum rate constant *k*_max_ = *k*_H_+*k*_0_.

## Results

### *c*NOR Proton Transfer Pathway 1

The K54^c^A and E58^c^Q variants from pathway 1 (K53^c^ and E57^c^ in *Ps*. *aeruginosa* and in Fig 1) that we previously constructed were specifically slowed in the proton-coupled electron transfer phase (ETPT, see Table 1 and Ref. [7]) during single-turnover reduction of O_2_ by the fully reduced *c*NOR. E58^c^Q was affected over the whole pH range. Therefore we now constructed an E58^c^D variant (which should be a milder substitution if the charge is more important than the structure) to further verify that this residue plays a role in proton transfer.

**Table 1:** Properties of *P*. *denitrificans c*NOR variants at the entrance of proton pathway 1. Data from this study is highlighted in bold, other data is from Ref. [7]. PW = pathway. Conservation and location from Ref. [7].

Res. in *P*. *aer*. (conservation) Location	Mutation in *P*. *den*.	Spectra	Maximum turnover activity with NO (e^-^/s) (% of WT activity) ^a)^	CO binding ^b)^ τ_1_ (μs)/ τ_2_ (μs) (% τ_2_) ^c)^	O_2_ ^b)^ binding τ (μs)	ETPT ^d)^ τ (ms) at pH 7.5
	WT *P*. *den*.	Normal	8.8 ± 0.2 (100%)	**14±4/130±10 (58±2%)**	**44 ± 2**	**17 ± 4**
E57^C^ (77% E, rest Q,H,D) Entrance proton PW 1	E58^C^Q *P*. *den*.	Normal	0.7 ± 0.0 (8%)	11**±**1/144**±**5 (49**±**1%)	42 **±** 2	**510 ±50**
E57^C^ (77% E, rest Q,H,D) Entrance proton PW 1	**E58**^**C**^**D *P*. *den*.**	**Normal**	**2.0 ± 0.2 (23%)**	**4±3/120±20 (62±**1**%)**	**70±10**	**102 ± 2**
K53^C^ (100%K) Entrance proton PW 1	K54^C^A *P*. *den*.	Normal	1.5 ± 0.1 (17%)	18**±**2/170**±**20 (62**±**2%)	40**±**2	250 ±80

a) At pH 7.5 and 303K. The average and the range of at least two different measurements is indicated.

b) At 1 mM CO or O_2_, pH 7.5 and T = 295K. The average of at least two independent experiments is given with the standard deviation (for wildtype, N>6 and E58^c^D and K54^c^A: N = 3) or with the range (N = 2).

c) % τ_2_ indicates the % of the total amplitude that is associated with the second phase at 430 nm.

d) Proton-coupled electron transfer during O_2_ reduction. The average of at least two independent experiments is given with the standard deviation (for wildtype, N = 12) or with the range (N = 2 or 3).

The E58^c^D variant could be expressed and purified and showed wildtype-like spectra. The variant had a higher multiple turnover activity with NO (23% of wildtype at pH 7.5 and 33% at pH 6.0) than the E58^c^Q (<10% of wildtype at pH 7.5 [7], see Table 1).

CO rebinding (after dissociation by a laser flash) was measured using flash photolysis. CO-rebinding is biphasic in wildtype *c*NOR [8,14] with rate constants and relative amplitudes that vary somewhat between preparations (see Table 1). Within the range of variation, the CO-binding properties are unchanged in the E58^C^D variant (see Table 1).

**Fig 2:**
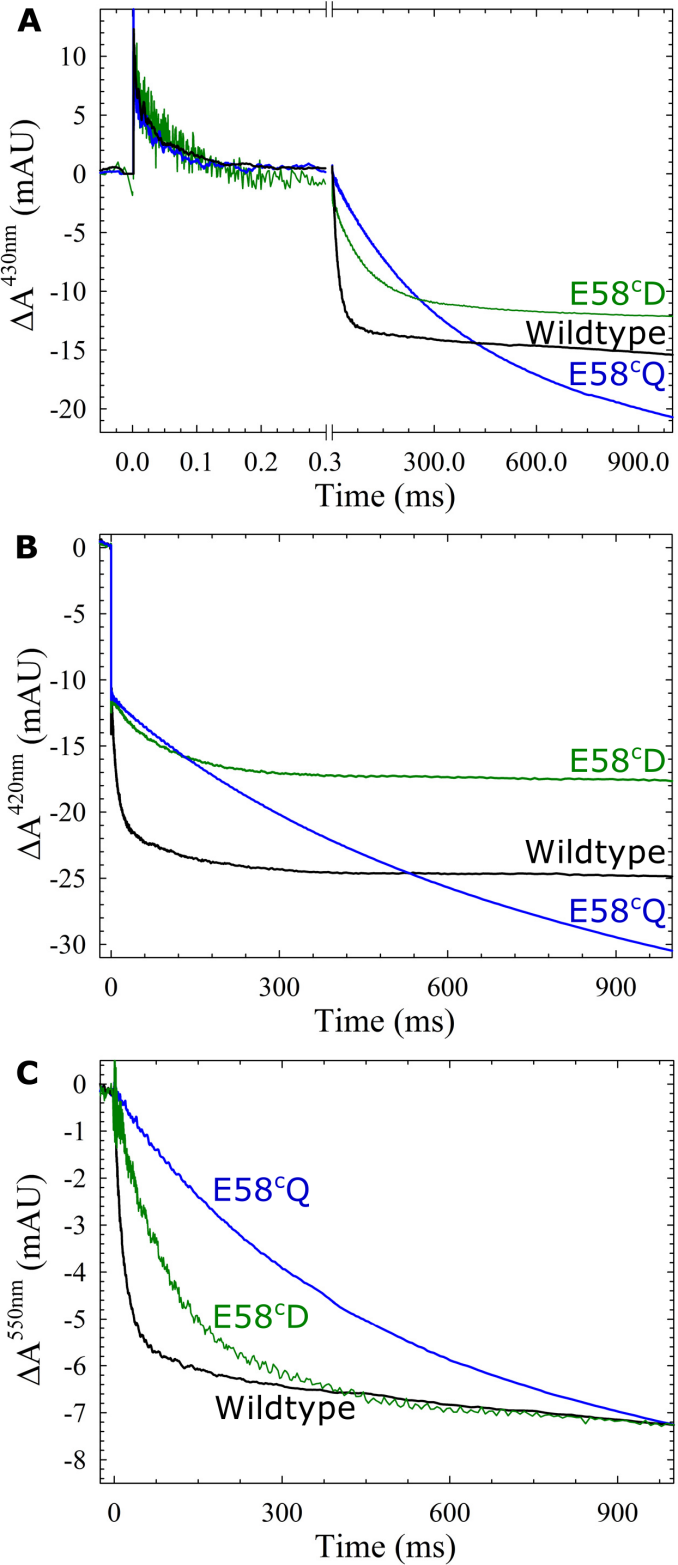
The reaction between fully reduced *c*NOR and O_2,_ showing the effect of changing the glutamate at the entrance of pathway 1. Fully reduced *c*NOR wildtype (black), E58^c^D (green) or E58^c^Q (blue) reacting with oxygen at pH 7.5. Traces show the absorbance change of *c*NOR as a function of time (with the laser flash dissociating CO at t = 0) at 430 nm (A), 420 nm (B) and 550 nm (C, reporting on the heme *c*). For clarity, the laser artefact at t = 0 has been truncated. At 420 nm and 430 nm the traces were normalized to the ΔA_CO,off_ step (variations occur in the amplitude of the ETPT, as observed before [7]). At 550 nm, the amplitudes at t = 1s were normalized to the same value in order to emphasize the changes in rate constants (the amplitude of E58^c^D was ~80% of wildtype). The data from wildtype and E58^c^Q are added for comparison from Ref. [7]. Experimental conditions: ~1 μM *c*NOR in 50 mM HEPES, pH 7.5, 50 mM KCl, and 0.05% (w/v) DDM, [O2] = 1 mM, T = 295K.

The single-turnover reaction between the fully reduced E58^C^D *c*NOR variant and oxygen was studied by flow-flash. At t = 0, CO is dissociated (the unresolved ΔA_COoff_ is visible at 420 nm and 430 nm as a rapid decrease and increase in absorbance, respectively, see Fig 2). In wildtype *c*NOR, we observe oxygen binding as a decrease in absorbance at 430 nm (mainly associated with changes at heme *b*_3_) with a time constant of 44 μs (see Fig 2A). This is followed by proton-coupled electron transfer (here called ETPT), causing a further decrease in absorbance at 430 nm with a time constant of 17 ms. This ETPT can also be observed as a decrease in absorbance at 420 nm (all hemes present in *c*NOR contribute at this wavelength, see Fig 2B) and 550 nm (reporting specifically on the heme *c*, only the ETPT is visible, see Fig 2C). Data for the E58^c^D at pH 7.5 are shown in Table 1 and Fig 2 together with the previous results [7] from E58^c^Q and wildtype for comparison.

**Fig 3:**
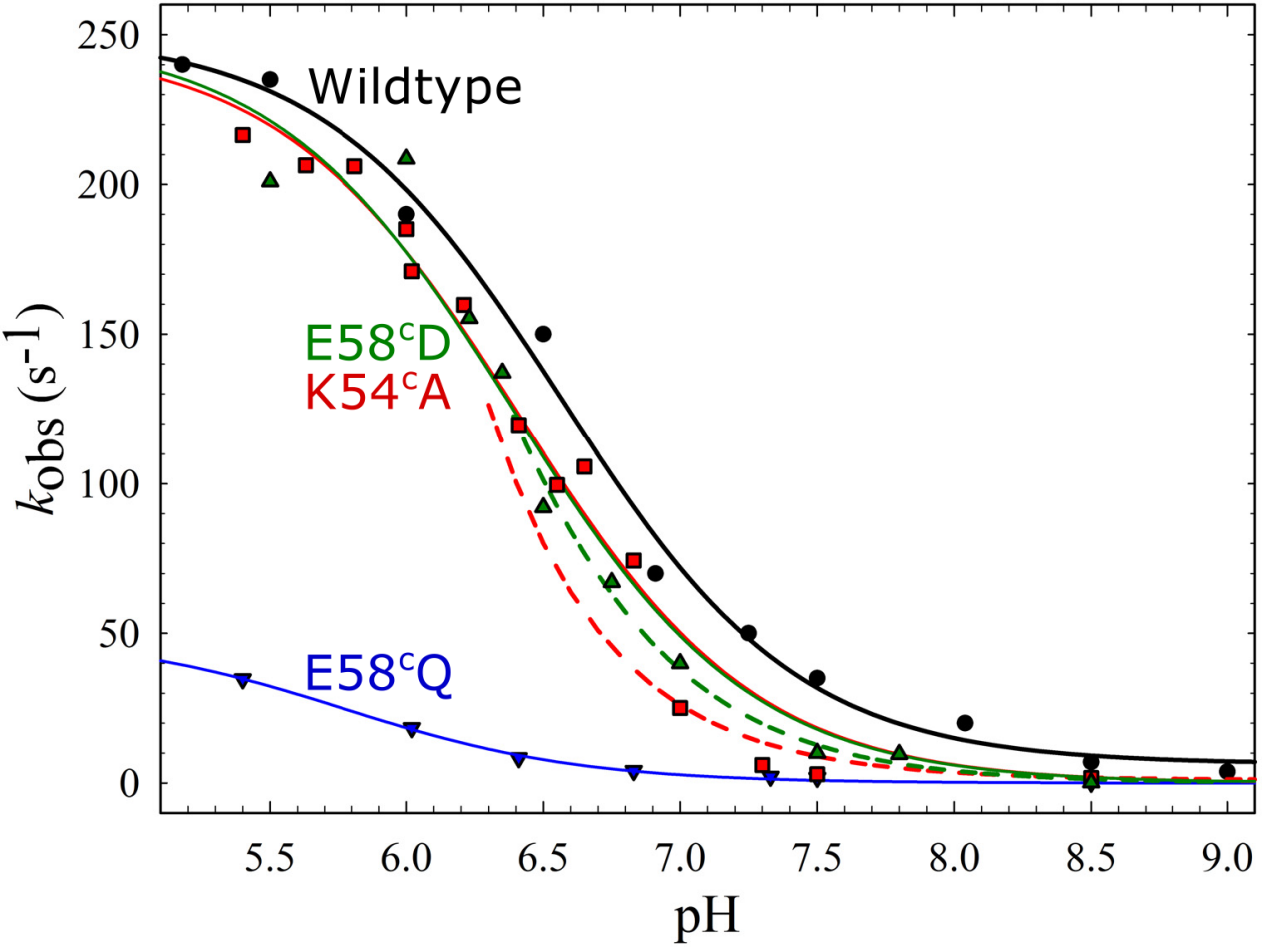
pH dependence of the proton-coupled electron transfer (ETPT) in the reaction between O_2_ and fully reduced *c*NOR variants at the entrance of proton transfer pathway 1. The rate constants of the ETPT plotted as a function of pH: wild type (black circles), K54^c^A (red squares), E58^c^Q (blue triangles down), E58^c^D (green triangles up). The data from E58^c^Q, K54^c^A and wildtype are from Ref. [7,8] (added for comparison). The data for wildtype was fitted to a pKa ~6.6 and a *k*_max_ (maximal rate at low pH) of ~250 s^-1^ (black line). The data for E58^c^Q was fitted to a p*K*a of ~5.8 and a *k*_max_ of ~50 s^-1^ (blue line, data and fit from from Ref. [7]). The data for K54^c^A was fitted to a p*K*a of ~6.4 and a *k*_max_ of ~250 s^-1^ (red line, data and fit from Ref. [7]) and for E58^c^D to a p*K*a of ~6.4 and a *k*_max_ of ~250 s^-1^ (green line). Note that data points for K54^c^A as well as the E58^c^D do not follow the fit around pH 7–7.5, and also plotted (see text) are two theoretical diffusion rate constants (*k*_diff_*[H^+^]) as a function of pH (= -log[H^+^]), assuming a *k*_diff_ of 2.5*108 M^-1^ s^-1^ in K54^c^A (red dashed line) and 3.5*108 M-1 s-1 in E58^c^D(green dashed line).

As can be seen in Table 1 and Fig 2A, O_2_ binding was ~1.5x slowed in the E58^c^D variant, while this was not the case for E58^c^Q. However the largest difference was that the E58^c^D variant was almost three times more active in multiple turnover and had a five times faster ETPT than E58^c^Q at pH 7.5. This indicates that the proton exchanging capability (or charge) of the residue plays a more important role than the length of the side-chain. However the E58^c^D variant still only had 23% activity and was around six times slower in ETPT when compared to wildtype at pH 7.5. To further investigate the ETPT, it was studied at various pH values and the fitted rate constants were plotted (see Fig 3).

It can be seen in S1 Fig that the pH dependence of NO turnover in *c*NOR wildtype is qualitatively very similar to the pH dependence of the ETPT. However, when we have made amino acid changes, we cannot know what reactions (ligand binding, NO reduction, electron input, proton uptake) are rate-limiting for turnover, so for assaying specific effects on proton transfer, we study the ETPT, which we know to be limited by proton transfer[7], in detail.

As can be seen in Fig 3 the maximal rate at low pH of E58^c^D reached similar values to wildtype (*k*max = *k*H + *k*0, fitted *k*H, E58^c^D = 250 ± 20 s-1 and *k*0 = 0 ± 10 s-1 versus *k*H, wildtype = 244 ± 7 s-1 and *k*0 = 6 ± 4 s-1, see also S1 Table). However the fitted p*K*a was shifted from 6.61 ± 0.05 for wildtype to 6.4 ± 0.1 for E58^c^D. Furthermore, just as previously noted in the K54^c^A variant [7], the simple one-p*K*a fit does not fit the data points at pH>7 for the E58^c^D variant (where rate constants are considerably slower than the model predicts). Therefore also plots of theoretical simple diffusion rate constants (*k’*_diff_ = *k*_diff_*[H^+^]) as a function of pH (= -log[H^+^]) that best fits the data points above pH 7 is shown in Fig 3 (*k*_diff_ = 3.5*10^8^ M^-1^ s^-1^ for E58^c^D and 2.5*10^8^ M^-1^ s^-1^ for K54^c^A, see also Discussion).

### Investigation of the role of residues in *c*NOR Pathway 2

The pathway 2 variants T67^c^V and E78^c^D (T66^c^ and E77^c^ in *Ps*. *aeruginosa* and Fig 1) could be expressed and purified and had wildtype-like spectra. Multiple turnover activities with NO were measured and compared to the activity of previously made variants in *P*. *denitrificans* and *T*. *thermophilus c*NOR (see Table 2).

**Table 2:** Properties *c*NOR variants in predicted proton pathway 2. Data from *T*. *thermophilus* is added for comparison from Ref. [13] and data from E78^c^F, Q394M and Q398L is from Ref. [7]. New data is highlighted in bold. Conservation and location from Ref. [7].

Res. In *Ps*. *aer*. (conservation) Location	Mutation in *P. den. /T. therm*.	Spectra	Maximum turnover activity with NO (% of WT activity) ^a)^	CO binding ^b)^ τ_1_ (μs)/ τ_2_ (μs) (% τ_2_) ^c)^ 0	O_2_ ^b)^ binding τ (μs)	ETPT ^d)^ τ (ms) at pH 7.5
	WT *P*. *den*.	Normal	8.8 ± 0.2 e^-^/s (100%)	**14±4/130±10 (58±2%)**	**44 ± 2**	**17 ± 4**
	WT *T*. *therm*.	Normal	5.5 ± 0.5 e^-^/min (100%)	-	-	-
T66^c^ (84% T, 15%S, rest:A) In conserved loop with Ca^2+^ ligands G71^C^ and Y73^C^	**T67**^**c**^**V *P*. *den*.**	**Normal**	**6.4 ± 0.2 e**^**-**^**/s (73%)**	**-**	**50±2**	**17 ± 2**
T66^c^ (84% T, 15%S, rest:A) In conserved loop with Ca^2+^ ligands G71^C^ and Y73^C^	T130^c^V *T*.*therm*.	Normal	0.77 ± 0.16 e^-^/min (14%)	-	-	**-**
E77^c^ (76%E,24%D) In conserved loop with Ca^2+^ ligands G71^C^ and Y73^C^, forms many hydrogen bonds.	**E78**^**c**^**D *P*. *den*.**	**Normal**	**5.1 ± 0.1 e**^**-**^**/s (57%)**	**3.9±0.1/133±2 (~46%)**	**43±5** ^e)^	**19 ± 3**
E77^c^ (76%E,24%D) In conserved loop with Ca^2+^ ligands G71^C^ and Y73^C^, forms many hydrogen bonds.	E78^C^F *P*. *den*.	This variant did not express				
E77^c^ (76%E,24%D) In conserved loop with Ca^2+^ ligands G71^C^ and Y73^C^, forms many hydrogen bonds.	D141^c^N *T*.*therm*.	This variant was not assembled				
E77^c^ (76%E,24%D) In conserved loop with Ca^2+^ ligands G71^C^ and Y73^C^, forms many hydrogen bonds.	D141^c^L *T*.*therm*^.^	Normal	0.99 ± 0.18 e^-^/min (18%)	-	-	-
Q411 **98% Q** (rest: E,D)	Q394M *P*. *den*.	Normal	5.9 ± 0.2 (67%)	16±1/145±2 (~57%)	38±1	18 ± 1
Q415 (71% Q, 21%E, 6%W, rest: S,V,Y)	Q398L *P*. *den*.	Normal	8.6 ± 0.4 (98%)	10±1/144±3 (56±3%)	31±9	21 ± 1

a) At pH 7.5 and T = 303K for *P*. *denitrificans*, at pH 6.0 and T = 315K for *T*. *thermophilus*. The average and the range of at least two different measurements is indicated.

b) At 1 mM CO or O_2_, pH 7.5 and T = 295K. The average is given with the standard deviation (wildtype, N>6) or the range between two different biological samples (Q398L). For T67^c^V, E78^c^D and Q394M the error of the fit is indicated based on ≥3 traces on the same sample.

c) % τ_2_ indicates the % of the total amplitude that is associated with the second phase at 430 nm.

d) Proton-coupled electron transfer during O_2_ reduction. The average is given with the standard deviation (wildtype, N = 12) or the range between two different biological samples (Q398L). For T67^c^V, E78^c^D and Q394M the error of the fit is indicated based on ≥3 traces at 420 nm, 430 nm and 550 nm of the same sample.

e) This number is the average of those obtained at pH 7, pH 6.75, and pH 8.5 because of a lack of reliable data at pH 7.5 (O_2_ binding was shown to be pH independent [8]).

As can be seen in Table 2, in *P*. *denitrificans c*NOR the variants in pathway 2 had wildtype rate constants for CO and O_2_ binding as well as considerable NO reduction activity. T67^c^V retained 73% of wildtype activity, whereas in the E78^c^D variant, activity was decreased to 57%. We therefore decided to further analyze the function of this *c*NOR variant in single turnover.

**Fig 4:**
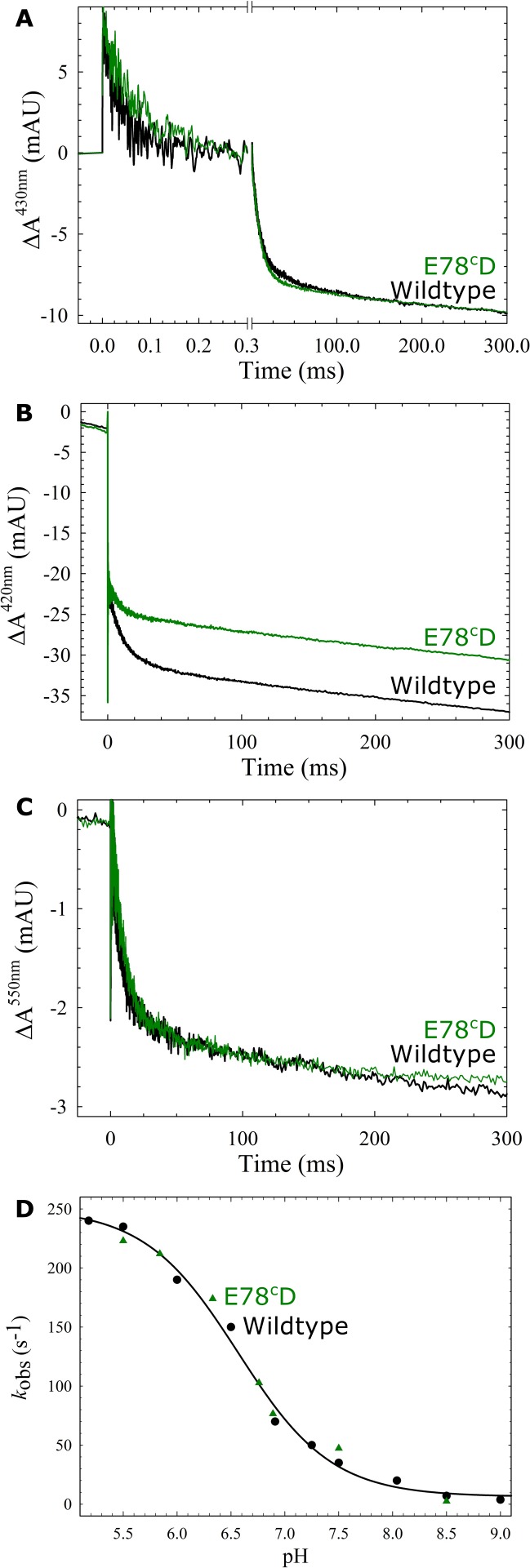
The reaction between fully reduced wildtype (black) or E78^c^D (green) *c*NOR and oxygen. Traces show the absorbance change of *c*NOR in time (with the laser flash at t = 0) at 430 nm (A), 420 nm (B) and 550 nm (C) at pH 7.5. Normalization as in Fig 2. The laser artefact at t = 0 is truncated for clarity. D) Rate constants of the proton-coupled electron transfer (ETPT) are shown as a function of pH. Rate constants for the E78^c^D variant are shown as green triangles. Wildtype rate constants (black circles) and fit (black line) are from Ref. [7,8]. A-C) Experimental conditions as in Fig 2.

The ETPT in the E78^c^D variant is very similar to wildtype (rate constants virtually identical, see Fig 4A and 4C). The only observed change in the ETPT for the E78^c^D variant is that the associated absorbance change at 420 nm is somewhat smaller (see Fig 4B), however we have observed variation in this amplitude previously [7,9]. We further studied the ETPT in this variant at several pHs, and found that the rate constants were comparable to wildtype at all pH values (see Fig 4D and S1 Table).

### Investigation of residues in the presumed continuation of the proton pathway

We then went on to exchange conserved residues in the region presumed to contain the continuation of the proton pathway towards the active site (Fig 1), with the aim to identify the proton donor with p*K*_a_ = 6.6. The exchanged residues were either direct calcium ligands (Fig 1: E135 and Y73^c^, see Table 3 for corresponding residues in the *P*. *denitrificans c*NOR), or located close to the calcium-binding site (Fig 1: E138, R134 and E70^c^) or close to the D-propionate of heme *b*_3_ (Fig 1: H339 and N335). Therefore the risk is higher that not only proton transfer is affected. We reconstructed the E122A and E125D variants (*P*. *denitrificans c*NOR numbering, first studied in Ref. [3]), purified E125D for the first time and re-purified E122A and E122D (first purified in Ref. [9]) with our current purification method.

**Table 3:** Properties of *c*NOR variants in the heme propionate and Ca^2+^ binding region. New data is shown in bold and compared to earlier published results from Refs. [3,7,9,11,13]. Conservation and location is taken from or calculated as described in Ref. [7].

Res. In *Ps*. *aer*. (conservation) Location	Mutation in *P. den. / T. therm*.	Spectra	Maximum turnover activity NO (% of WT activity) ^a)^	CO ^b)^ binding τ_1_ (μs)/ τ_2_ (μs) (% τ_2_) ^c)^ 0	O_2_ ^b)^ binding (μs) 0	ETPT ^d)^ τ (ms) at pH 7.5
	WT *P*. *den*	normal	100%	**14±4/130±10 (58±2%)**	**44 ± 2**	**17 ± 4**
E70 ^C^ (80% E, rest N,H,Q,S,F) In conserved loop with Ca^2+^ ligands	**E71**^**C**^**Q *P*. *den***	**α-region slightly altered** ^**e)**^	**as background 0%**	**13±1/157±1 (67±1%)**	**85 ± 4**	**-**
E70 ^C^ (80% E, rest N,H,Q,S,F) In conserved loop with Ca^2+^ ligands	**E71**^**C**^**D *P*. *den***	**α-region altered** ^**e)**^ **less pure**	**27±3% not sigmoidal**	**40±20/160±20 (50±9%)**	**48 ± 4**	**(8 ± 1)**^f)^
Y73^C^ (99% Y, rest R) Ca^2+^ ligand	**Y74**^**C**^**S *P*. *den***	**α-region slightly altered** ^**e)**^	**53±3%**	**14±10/150±30 (55±1%)**	**43 ±3**	**13 ± 2**
Y73^C^ (99% Y, rest R) Ca^2+^ ligand	**Y74**^**C**^**F *P*. *den***	**normal**	**53±3%**	**16±1/121±2 (55±1%)**	**48 ±7**	**29 ± 1**
R134 (96%, rest NIML) Next to Ca^2+^ ligand E135	**R121Q *P*. *den***	**normal**	**9.4±0.3%**	**30±10/200±20 (60±10%)**	**50 ± 7**	**(32± 7)**^f)^
E135 (88%E,9%K, rest: H,S,V,P) Ca^2+^ ligand	**E122A *P*. *den*.**	**normal**	14% **35±6%**	5–10/50–100 (~50%) **19±4/140±10 (57±3%)**	40, **55±5**	**17 ± 1**
E135 (88%E,9%K, rest: H,S,V,P) Ca^2+^ ligand	**E122D *P*. *den*.**	**normal**	83% **30±8%**	5–10/50–100 (~50%) **30±10/170±30 (52±6%)**	~40, **80±10**	~10 **(30±10)**^f)^
E135 (88%E,9%K, rest: H,S,V,P) Ca^2+^ ligand	E122Q *P*. *den*.	normal	1%	5–10/50–100 (~50%)	~40	-
E138 (96%rest: D) In loop with Ca^2+^ ligand	**E125D *P*. *den***	**altered less pure**	2%**, <10%**	**30±8/180±20 (62±4%)**	**47±2**	**-**
E138 (96%rest: D) In loop with Ca^2+^ ligand	E125Q *P*. *den*	-	13%	-	~40	-
E138 (96%rest: D) In loop with Ca^2+^ ligand	E125A *P*. *den*	normal	4–7%	-	~40	-
D198 (96%D 2%E, rest: A,S,Q) In proton transfer PW1, H-bonds with R134, K53^C^	D185E *P*. *den*	normal	12±1%	29±1/146±6 (54±2%)	52 ± 4	**(33±2)**^**g)**^
D198 (96%D 2%E, rest: A,S,Q) In proton transfer PW1, H-bonds with R134, K53^C^	D185N *P*. *den*	normal	<<10%	21±1/147±1 (~58%)	62 ± 1	**(18±6)**^g)^
D198 (96%D 2%E, rest: A,S,Q) In proton transfer PW1, H-bonds with R134, K53^C^	D185A *P*. *den*	normal	as background 0%	34±1/145±2 (~51%)	64 ± 3	-
N335 (100% N) H-bond D-prop. heme *b*_3_	**N322L *P*. *den***	**altered** ^**e)**^	**51±6%**	**24±6/180±10 (70±10%)**	**58 ± 4**	**(21±4)**^f)^
N335 (100% N) H-bond D-prop. heme *b*_3_	N335L *T*. *therm*.	normal ^h)^	15%	N.D.	N.D.	-
H339 (99%H, 1xR) H-bond D-prop. heme *b*_3_	**H326F *P*. *den***	**altered** ^**e)**^	**as background 0%**	**58±1/178±4 (39±2%)**	**40 ± 3 (+slower fraction)**	**-**
H339 (99%H, 1xR) H-bond D-prop. heme *b*_3_	H339F *T*. *therm*.	normal ^h)^	0%	N.D.	N.D.	-

a) At pH 7.5 and T = 295K for *P*. *denitrificans*, at pH 6.0 and T = 315K for *T*. *thermophilus*. For new data the average and the range of at least two different measurements is indicated.

b) At 1 mM CO or O_2_, pH 7.5 and T = 295K. The average is given with the standard deviation (wildtype: N>6, E122A, N322L and E71^c^D: N = 3) or the range between two different biological samples, except for D185N and E71^c^Q where the error of the fit is indicated based on ≥3 traces on the same sample.

c) % τ_2_ indicates the % of the total amplitude that is associated with the second phase at 430 nm.

d) Proton-coupled electron transfer. The average is given with the standard deviation (wildtype: N = 12, N322L and E71^c^D: N = 3) or the range between two different biological samples except for D185N where the error of the fit is indicated based on ≥3 traces at 430 nm and 550 nm of the same sample.

e) Especially after incubation with CO, see S2 Fig.

f) The fit of these variants is based only on the 430 nm and 550 nm data, since they did not have any signal for the ETPT at 420 nm.

g) Not fitted in Ref. [7] because of absence signal for ETPT at 420 nm and small signals at 430 nm and 550 nm. Now fitted with 550 nm and 430 nm data as described for the other variants in this table.

h) Oxidized and reduced spectra only [13].

All variants could be expressed and purified, but the amount and purity of E71^c^D and E125D (E70^c^ and E138 in *Ps*. *aeruginosa* numbering) was considerably lower than for wildtype (A_280nm_ /A_410nm_ ratio ~2.3 for E71^c^D and ~2.1 for E125D instead of 1–1.5 for wildtype). E125D also had altered spectra as compared to wildtype (see S2 Fig). For various other variants the absorbance spectra were also altered in the alpha region after incubation of the reduced variant with CO (see Table 3 and S2 Fig for all spectra). H326F (H339 in *Ps*. *aeruginosa* and Fig 1) had the largest change, and also showed changes in the 560 nm region already in the reduced spectrum. Changes after CO incubation were also seen for N322L (N335 in *Ps*. *aeruginosa* and Fig 1), E71^c^Q and E71^c^D (E70^c^ in *Ps*. *aeruginosa* and Fig 1). The alteration of the spectra was more severe for E71^c^D than for E71^c^Q. Some changes were also seen for Y74^c^S over time, but changes were minimal in Y74^c^F (Y73^c^ in *Ps*. *aeruginosa* and Fig 1). Since the peak position of the *c*NOR variants in the CO bound form in the Soret region does not change and no additional peaks appear, CO presumably still only binds to heme *b*_3_ in these variants, but seems to cause a larger change in conformation over time. R121Q (R134 in *Ps*. *aeruginosa* and Fig 1) showed wildtype like spectra, also after overnight incubation with CO. Also the spectra of E122A/D were comparable to wildtype (see S2 Fig).

#### Multiple turnover

As can be seen in Table 3, multiple variants had no (E71^c^Q, H326F) or less than 10% (R121Q, E125D) multiple turnover activity at pH 7.5 compared to wildtype. Also at pH 6.0 (where the *c*NOR wildtype activity is higher, see S1 Fig and previously shown for the *P*. *aeruginosa* enzyme in Ref. [16]), H326F and E71^c^Q had no activity above background. Surprisingly the N322L variant still showed ~50% of wildtype activity at pH 7.5 (~65% at pH 6.0), even though the asparagine is completely conserved and the CO-bound spectra were altered. The E122A variant had 2.5x higher turnover activity than previously reported [3], while the E122D had almost 3x lower turnover (see Table 3). The low activity for E125D was in agreement with previously reported data. It should be noted that previously reported multiple turnover rates were measured in *E*. *coli* membranes as the initial rate at higher [NO] [3,11], whereas we report the turnover activity as the maximum observed rate constant at lower [NO], which might be a reason for differences.

**Fig 5:**
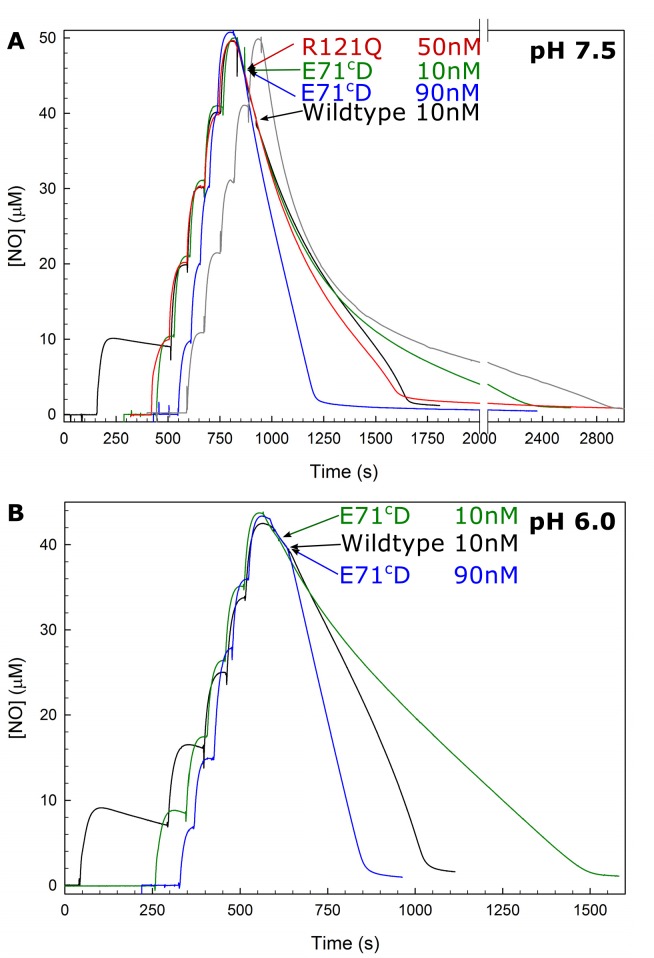
Multiple turnover activity of *c*NOR variant E71^c^D with NO is not sigmoidal. The NO reduction by the E71^c^D variant is shown at two concentrations: 10 nM (green line) and 90 nM (blue line) at pH 7.5 (A), and at pH 6.0 (B). For comparison data for 10 nM wildtype (black line), 50 nM R121Q (red line, only in A), and background consumption (no enzyme added, grey line in A) is also shown. R121Q has a lower multiple turnover activity, but is still sigmoidal. Experimental conditions: T = 295K, NO-saturated water (2 mM NO) was added in five steps of 5 μl (10 μM/addition) to a deoxygenated solution of 50 mM HEPES, pH 7.5, 50 mM KCl, 0.05% (w/v) DDM, 30 mM glucose, 20 units/ml catalase, 1 unit/ml glucose oxidase, 500 μM TMPD, and 20 μM horse heart cytochrome *c*. Then 3 mM ascorbate was added (giving some background NO reduction), followed by *c*NOR addition (as indicated by arrow and label). Note that the background trace shown in A was recorded at 303K, whereas the traces with *c*NOR were recorded at 295K, which explains the high background consumption at high [NO].

The E71^c^D variant had a significant turnover activity with NO (27% compared to wildtype at pH 7.5 and 21% at pH 6.0), but did not show the sigmoidal behaviour indicative of substrate inhibition that is normally seen for *c*NOR (see Fig 5). This effect is not due to progressive degradation of the variant during turnover, since the observed NO reduction rates are still linear with protein concentration (S3 Fig). This behaviour would not be expected if the lack of sigmoidal behaviour was due to progressive degradation since we report the turnover activity as that at low [NO] shortly before depletion, and at higher enzyme concentrations, each enzyme molecule will have undergone fewer turnovers in total.

#### CO and O_2_ binding

**Fig 6:**
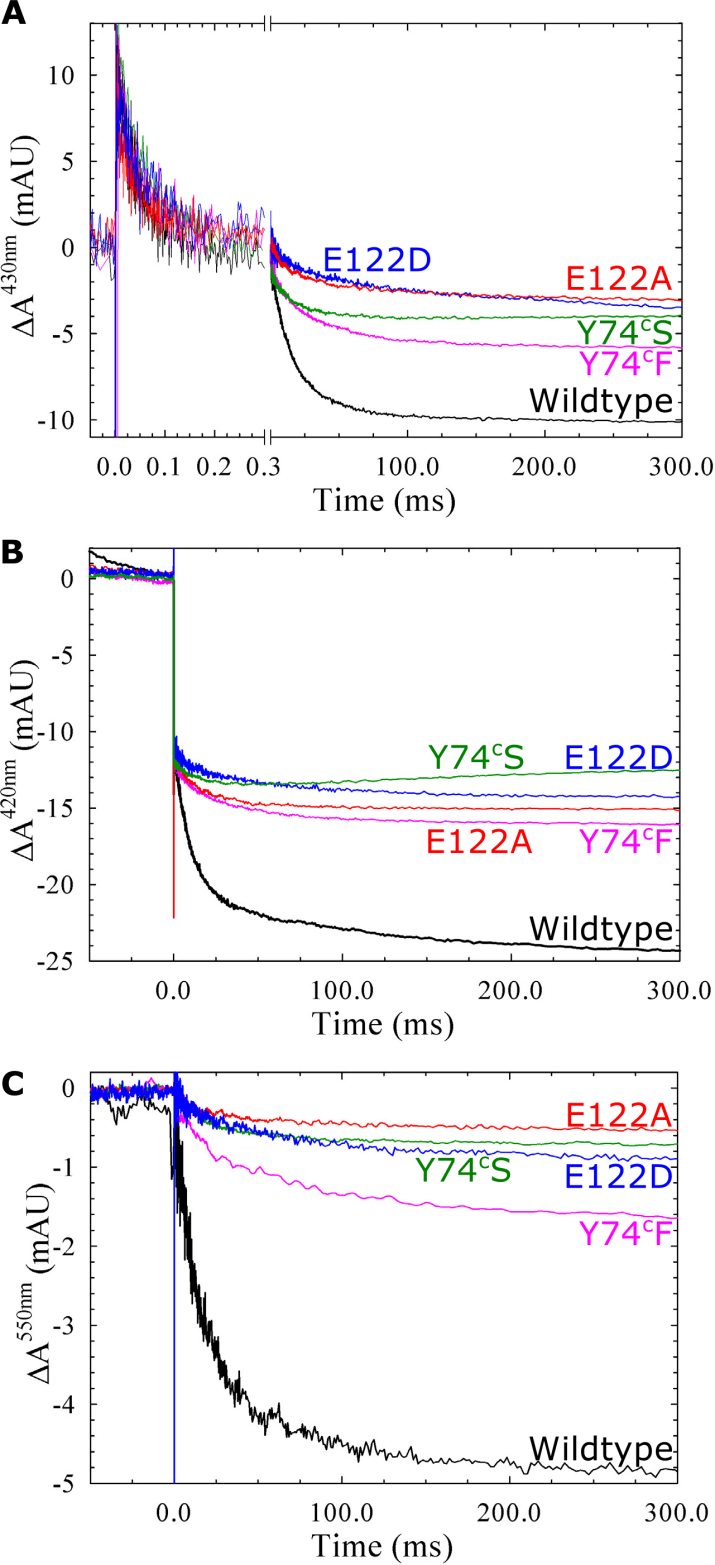
Reaction between oxygen and fully reduced *c*NOR wildtype or variants of calcium ligands E122 and Y74^c^. Traces show the absorbance change of *c*NOR in time (with the laser flash at t = 0, which gives a short artefact) at 430 nm (A), 420 nm (B) and 550 nm (C, reporting on the heme *c*). At 420 nm and 430 nm the traces were normalized to the ΔA(CO_off_), at 550 nm the traces were normalized as at 420 nm. Experimental conditions as in Fig 2.

**Fig 7:**
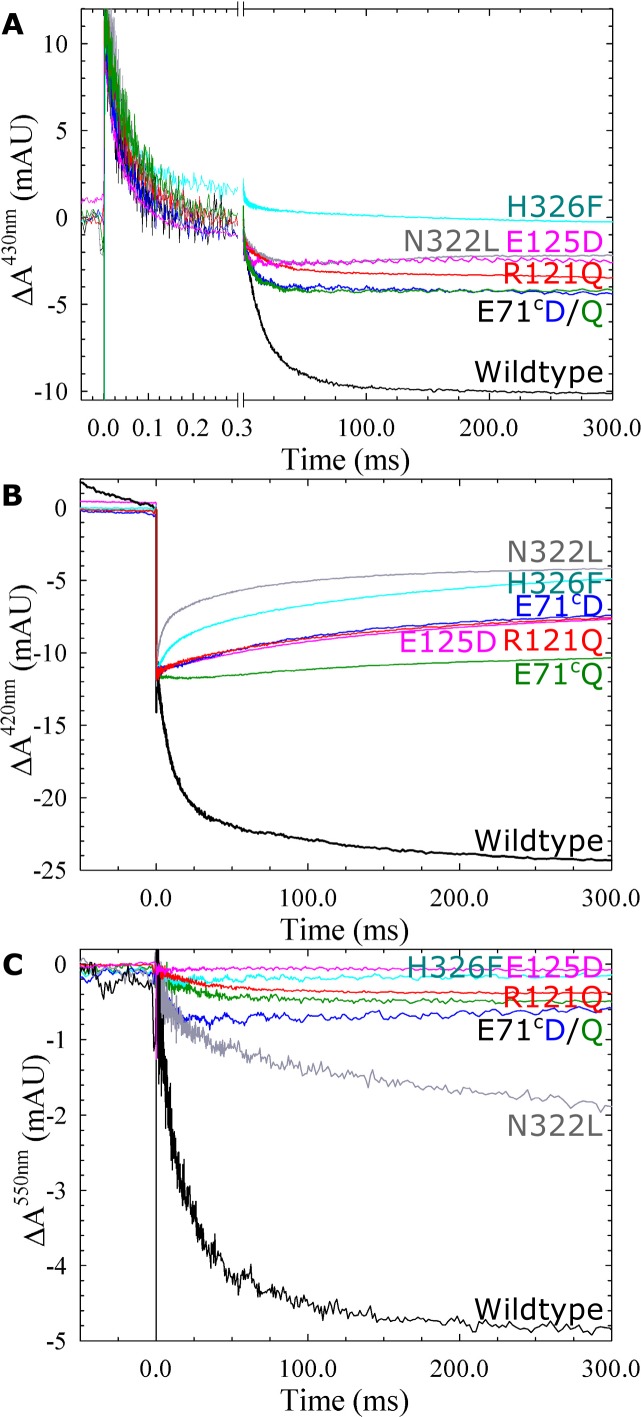
Reaction between fully reduced *c*NOR wildtype and variants with oxygen. The variants have changes around the calcium site (R121Q, E125D, E71^c^Q, E71^c^D) or around the D-propionate of heme *b*_3_ (N322L and H326F). Traces show the absorbance change of *c*NOR in time (with the laser flash at t = 0, which gives an artefact) at 430 nm (A), 420 nm (B) and 550 nm (C, reporting on the heme *c*). Traces normalized as in Fig 6, experimental conditions as in Fig 2.

CO rebinding after photolysis of the fully reduced CO-bound *c*NOR variants was slowed significantly in H326F, E71^c^Q/D, E125D, R121Q and N322L (see Table 3; for E71^c^Q and N322L the percentage of the slow phase (τ_2_) is larger, for E71^c^D the τ_1_ is larger, for R121Q, E125D, N322L and H326F both τ_1_ and τ_2_ are larger). The Y74^c^S/F variants had wildtype-like CO rebinding rates as did the E122 variants (in agreement with what had been described earlier [9]). O_2_ binding, studied in the flow-flash reaction between the fully reduced CO-bound *c*NOR variants and O_2_ (see Table 3 and Figs 6 and 7), is largely unaffected (at a τ of 44 μs) in most variants (Table 3). The E71^c^Q variant shows significantly slower O_2_ binding (τ = 85 μs), and in the H326F variant there is a fraction that binds O_2_ slowly. O_2_ binding is also slowed, but to a smaller extent in the N322L variant (τ = 58 μs) and E122A (τ = 55 μs). The E122D variant also showed slowed O_2_ binding (τ = 80 μs) which was not observed previously [9].

#### Proton-coupled electron transfer (ETPT) during single-turnover with O_2_

For all variants, we also studied the ETPT during the reaction between fully reduced *c*NOR variants and O_2_ (Figs 6 and 7). We remeasured the *c*NOR Ca^2+^-ligand variants E122A and E122D, but we could not reproduce the wildtype-like amplitudes for E122D [9]. However we observed a larger amplitude for the ETPT in the E122A variant than before [9]. The amplitudes varied significantly between preparations, and when we left out EDTA from the purification, the amplitudes generally got somewhat larger for E122D (see S4 Fig). We think this discrepancy is due to partial disruption of the Ca^2+^ site in this variant and that the degree to which Ca^2+^ is lost is highly variable (see Discussion).

The H326F, that was inactive in multiple turnover, did not show any ETPT signal (only oxygen binding at 430 nm was observed, Fig 7A). The other variants showed signals for the ETPT reaction at 430 nm and 550 nm, even the E71^c^Q that had no multiple turnover activity above background. However, only the E122 and Y74^c^ variants showed signals for ETPT also at 420 nm (Fig 6B). For the other variants only the CO_off_ step and what we presume is CO rebinding (with positive amplitude, see Discussion) is seen at this wavelength (Fig 7B), even for those with significant multiple turnover activity (such as E71^c^D and N322L). Also for the E122A/D and Y74^c^S/F variants the 420nm-amplitude was much smaller than for wildtype (where the ETPT step is usually as large as the CO_off_ step although variation occurs, see Fig 6B). In the Y74^c^ variants the ETPT signals increased with decreasing pH but the 420nm-signal was absent at pH values of 8 and higher (see S5 Fig and Discussion).

**Fig 8:**
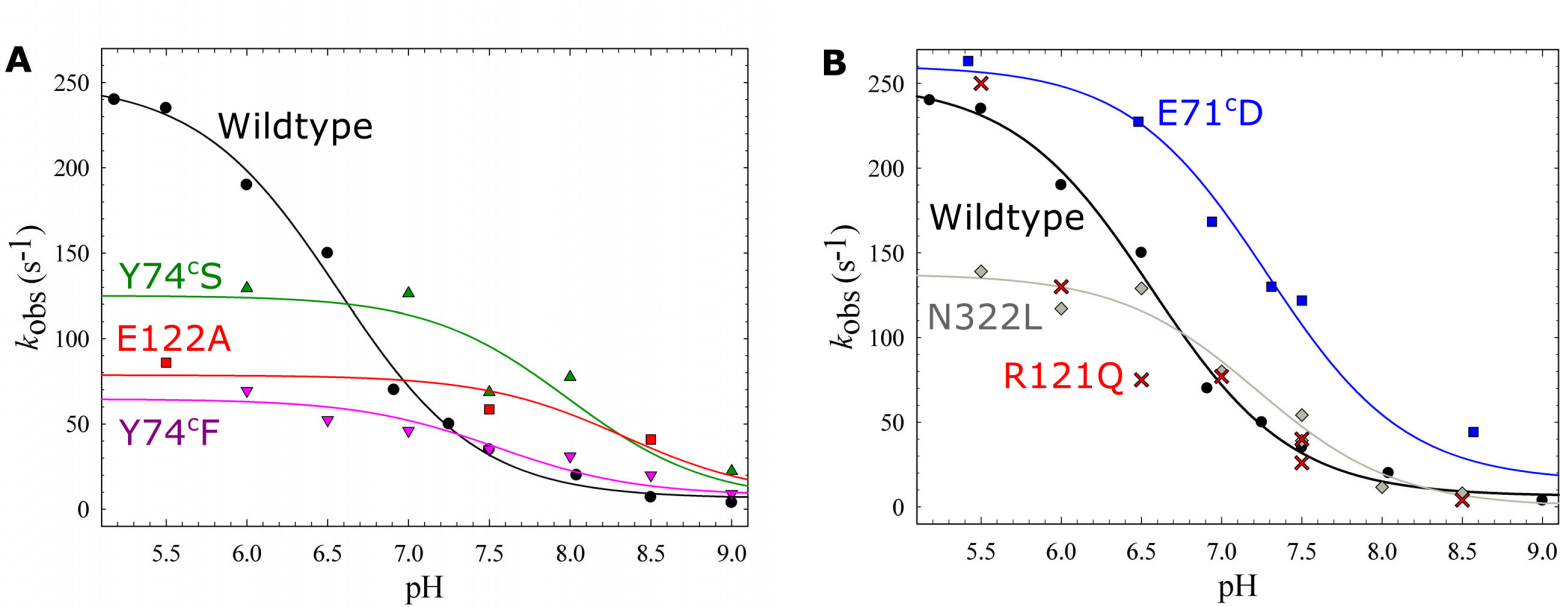
pH dependence of the ETPT for *c*NOR variants in and around the Ca^2+^ binding site and heme propionate region. A) Variants in the calcium ligands E122 and Y74^c^ compared to wildtype (black circles). Y74^c^S (green triangles up), Y74^c^F (pink triangles down), E122A (red squares). Shown are also the fits described in the text: Y74^c^S (green line, p*K*_a_~8.0), Y74^c^F (pink line, p*K*_a_~7.5), E122A (red line, p*K*_a_~8.3). B) ETPT rate constants tentatively determined for variants with small amplitudes of ETPT, compared to wildtype (black circles). N322L (grey diamonds) and R121Q (approximate fits shown by red crosses) and E71^c^D (blue squares). Shown are also the fits described in the text; E71^c^D (blue line, p*K*_a_~7.3), N322L (grey line, p*K*_a_~7.2). A,B) The WT data (from [7,8]) was fitted to a p*K*_a_ of ~6.6 (black line).

For all *c*NOR variants that showed activity in multiple turnover with NO, we also studied the pH dependence of the ETPT reaction (see Fig 8).

For the E122 and Y74^c^ variants the ETPT rate constants were fitted at each pH by using the 420 nm, 430 nm and 550 nm traces (see Fig 8A). For the other variants (see Fig 8B) the 420 nm traces were not used since they showed no amplitude for the ETPT. It was difficult to fit the ETPT rate of the R121Q samples reliably, and no p*K*_a_ was determined, but the variant still showed a pH dependence with rates similar to wildtype (see Fig 8B). Wildtype has a p*K*_a_ of 6.6±0.1, a *k*_H_ of 244±7 s^-1^ and a *k*_0_ = 6±4 s^-1^ (*k*_max_ = *k*_H_+*k*_0_ = 250 s^-1^, see Materials and Methods) [8].

The variants in the calcium ligands E122 and Y74^c^ all showed a much smaller pH dependence in the studied range (Fig 8A). Y74^c^S was fitted with p*K*_a_ = 8.0±0.4, *k*_H_ = 120±30 s^-1^ and *k*_0_ = 5±30 s^-1^ (*k*_max_ = 125 s^-1^). Y74^c^F was fitted with p*K*_a_ = 7.5±0.2, *k*_H_ = 57±7 s^-1^ and *k*_0_ = 8±6 s^-1^ (*k*_max_ = 65 s^-1^). A tentative fit for E122A gave p*K*_a_~8.3, *k*_H_~70 s^-1^ and *k*_0_~10 s^-1^ (*k*_max_~80 s^-1^), but this is only approximate because of the limited amount of points.

The pH dependence was also altered for N322L and E71^c^D (close to the D-propionate of the *b*_3_ heme and the calcium binding site, respectively, see Figs 1 and 8B). N322L was fitted with p*K*_a_ = 7.2±0.1, *k*_H_ = 138±7 s^-1^ and *k*_0_ = 0±6 s^-1^. The fitted E71^c^D rates were faster than wildtype; the p*K*_a_ shifted to 7.3±0.1, *k*_H_ = 250±20 s^-1^, *k*_0_ = 15±20 s^-1^ (*k*_max_ = 265 s^-1^, similar to wildtype). An overview of all fitted p*K*_a_ and *k*_max_ values can be found in S1 Table.

## Discussion

### Proton entry

Based on the crystal structure and molecular dynamics simulations, three putative proton transfer pathways leading from the periplasmic bulk into the active site in *c*NOR were predicted [6,17]. We previously reported that out of these, only the proton pathway 1 is used in the *c*NOR from *P*. *denitrificans* [7]. This was largely based on the behavior of the K54^c^A and E58^c^Q (K53^c^ and E57^c^ in *Ps*. *aeruginosa c*NOR, see Fig 1) variants that have a substitution at the entrance to pathway 1 at the surface. These variants were specifically slowed in the proton-coupled electron transfer phase (ETPT) during reduction of oxygen by the fully reduced *c*NOR. In this study, we constructed the E58^c^D variant to further verify that this residue plays a role in proton transfer. This should be a milder substitution if protonation is important, but a more severe one if the glutamate plays a structural role as Asp is one methyl group shorter than Glu. Our results show that catalytic NO-reduction activity in the E58^c^D variant is higher than in E58^c^Q (Table 1). Furthermore, the maximal rate of the ETPT at low pH in the E58^c^D variant was similar to that in wildtype (*k*_max_~255 s^-1^) whereas the *k*_max_ was below 50 s^-1^ for E58^c^Q (see Fig 3 and S1 Table). The E58^c^D variant was thus significantly less affected than E58^c^Q, supporting that this residue indeed plays a role specifically in proton transfer. The p*K*a of the ETPT was only slightly shifted from ~6.6 in wildtype to ~6.4 in E58^c^D. However, this fit to a single p*K*a did not fit well to the data at pH > 6.5, similar to the situation in the previously described K54^c^A variant ([7], see Fig 3). We previously interpreted this as a break-down of the assumption in the model (Eqs 1 and 2) that there is a rapid equilibrium between the internal proton donor (AH) and the outside bulk pH. Thus at higher pH for the K54^C^A, proton diffusion to the donor AH instead becomes rate-limiting, and the pH dependence is better fitted by a linear relation between the observed rate constant and the [H^+^] (the plot in the figure is not linear since it shows the dependence on pH). This interpretation is strengthened by the similar behavior of the E58^c^D variant (Fig 3), in which this (fitted) diffusion is somewhat faster (*k*_diff_ ~3.5*10^8^ M^-1^ s^-1^) than in K54^c^A (*k*_diff_ ~2.5*10^8^ M^-1^ s^-1^). Presumably, this diffusion rate constant from the bulk to the proton donor is much faster in wildtype (maximum values for proton diffusion in proteins are >10^10^ M^-1^s^-1^, see e.g. [18]). Thus, the E58^c^D and K54^c^A alterations both lead to strikingly similar proton-transfer properties, even though the effect on e.g. local electrostatics should be significantly different. In the MD simulations [6], the equivalents of the E58^C^ and K54^C^ (in the *Ps*. *aeruginosa c*NOR) form a salt bridge at the very entrance to the path. This bridge opens occasionally, letting a water-assisted continuous hydrogen-bonded chain form. This suggested ‘gating’ role for these amino acids in proton entry is supported by our data both in terms of the importance of E58^c^ and K54^c^, and in terms of the similar effect observed when the gate dynamics/equilibration is changed by either alteration. That the milder Glu58^C^-Asp exchange has a somewhat less severe effect on the rate of diffusion than the Lys54^C^-Ala additionally supports the proton transfer role of this site.

In a study on the *c*NOR from *T*. *thermophilus* it was concluded that it was not likely that pathway 1 was used for proton transfer [13]. This was based in part on the observation of the wildtype-like activity when D198 (in both *T*. *thermophilus* and *Ps*. *aeruginosa* numbering) was exchanged for a glutamate or asparagine. Instead, several pathway 2 variants (T130^C^V, D141^C^L/N and D209^C^L/E/N) were reported to be significantly affected in multiple turnover [13]. Therefore, in order to further explore the possibility that pathway 2 is also used in the *P*. *denitrificans c*NOR, we constructed variants as similar as possible to these. Since E145^c^ in *Ps*. *aeruginosa c*NOR (D209^c^ in *T*. *thermophilus c*NOR*)* is an alanine in the *P*. *denitrificans c*NOR (A149^c^), we focused on the other two residues of the study (*Ps*. *aeruginosa* numbering, with numbering from *T*. *thermophilus* (*Tt*) and *P*. *denitrificans (Pd)* between brackets); T66^c^ (*Tt*: T130^C^, *Pd*: T67^c^) and E77^c^ (*Tt*: D141^C^, *Pd*: E78^c^). The threonine was changed to a valine (*Pd*: T67^c^V) as the T130^c^V *T*. *thermophilus c*NOR variant. The glutamate, E77^c^, is in the loop with the calcium ligand Y73^c^ (see Fig 1) and the charge is conserved (76% E and otherwise D) [13]. The previously made *P*. *denitrificans c*NOR E78^c^F variant did not express (Table 3 and [7]). We therefore decided to make a milder substitution, the E78^c^D, while the reported *T*. *thermophilus c*NOR variants were D141^c^N (not assembled) and D141^c^L (only 18% active) [13]. As can be seen in Table 2, the *P*. *denitrificans c*NOR variants in pathway 2 were not nearly as affected as the described *T*. *thermophilus c*NOR variants. T67^c^V retained 73% activity compared to only 14% in *Tt c*NOR. In the case of E78^c^D, we did observe a decrease in activity to 57%. However, the rate constants for the ETPT during O_2_-reduction (which is limited by proton transfer [7]) in the E78^c^D variant were very similar to wildtype over the whole pH range (see Fig 4). We therefore conclude that the rate of proton transfer is unaffected in this variant, and the slowing of turnover has other reasons, possibly heterogeneity (manifested in the smaller amplitude of the ETPT at 420 nm, see Fig 4) or a slowing of re-reduction. We note that the E78^c^D is a very mild substitution but that the same Glu-Asp exchange at the E58^C^ in pathway 1 showed very significant effects on proton transfer dynamics.

The data presented here thus further strengthens the evidence that only pathway 1 is used for proton transfer in *c*NOR from *P*. *denitrificans*. We cannot exclude that (an)other pathway(s) is/are used in more distantly related *c*NORs (such as the one from *T*. *thermophilus*), but we think that *c*NORs belonging to the proteobacteria phylum [13] (to which the crystallized *c*NOR from *Ps*. *aeruginosa* also belongs) use only proton pathway 1.

### Variants in the continuation of the proton pathway and the presumed proton donor

#### General properties of constructed variants

It turned out to be more difficult to determine the continuation of the proton transfer pathway and to identify the proton donor. As could be expected, changes closer to the active site started to interfere with *c*NOR stability and the incorporation/stability of the co-factors. For wildtype *c*NOR the fully reduced CO-bound species is very stable over time as evidenced by the optical spectra, where only the 420 nm peak increases somewhat over time (related to slow full reduction of the low-potential *b*_3_ heme). However the optical changes upon CO binding are much larger in some variants and they increase further over time for the E71^c^D/Q, Y74^c^S, N322L and H326F variants (see S2 Fig), indicating a slow change in the conformation of the CO-bound heme *b*_3_. This change is most severe in H326F, substantial in N322L and E71^c^D and also observed but less severe in E71^c^Q and Y74^c^S. The spectrum of the E125D variant was altered under all conditions (see S2 Fig). We note however, that several *c*NOR variants (R121Q, E122A, and Y74^c^F, see S2 Fig) show basically unaltered optical spectra. The heme *c* peak at 551 in the reduced spectra is basically unaltered in all studied variants (the changes in the *c*NOR variants are also far away from the *c* heme, see Figs 1 & S1). We note that the variants with the largest spectral changes (H326F and N322L) are directly hydrogen-bonding to the *b*_3_ heme propionate D, whereas variants in the Ca^2+^-ligands (E122, Y74^c^) do not show severe changes in the optical spectra. We thus think the changes are more likely due to conformational changes to heme *b*_3_ itself than to a loss of the Ca^2+^. The Ca^2+^-site coordinates the A-propionate of *b*_3_ (see Fig 1) and therefore variants around the Ca^2+^-site could also have altered spectra due to changes in the heme *b*_3_.

#### NO inhibition in multiple turnover

The multiple turnover activity of wildtype *c*NOR with NO shows a sigmoidal behaviour (Fig 5) caused by substrate inhibition at high [NO]. This behaviour was suggested to be due to NO binding to the oxidized enzyme [19], and it is more pronounced when the electron mediator concentration is low [20], which could be due to slow rereduction of the non-productive NO complex [20]. However, NO inhibition is observed already in single turnover (without external re-reduction) of the fully reduced *c*NOR with NO [10]; transfer of electrons from the hemes *c* and *b* (corresponding to the ETPT described above) to the active site was faster at 75 μM than at 1.5 mM NO. It has been suggested from calculations that inhibition by NO is due to NO binding to the μ-oxo bridge in the oxidized active site forming nitrite at the heme *b*_3_ and reducing Fe_B_ [21]. In the theoretical study, the non-productive nitrite complex could revert to the μ-oxo bridged state and continue on the catalytic cycle from there. That the NO-bound form of the oxidised *c*NOR is not a ‘dead-end’ complex is consistent with the kinetic study [10] where only the rate and not extent of the reaction was affected by [NO]. Wherever the binding site is located, it is specific to NO since there is no substrate inhibition with O_2_.

In the E71^c^D variant the substrate inhibition with NO is not observed (Fig 5). We varied both the *c*NOR/NO ratio and the pH, but never observed a sigmoidal behaviour for this variant. The loss of the NO inhibition could readily be explained if the multiple turnover rates were so slow that the inhibitory effect of NO does not affect the rate-limiting step. However, R121Q has a much lower turnover activity (9 versus 27% of wildtype) and still shows the sigmoidal behaviour (see Fig 5A). The E71^c^D variant could also undergo a progressive decrease in activity (so-called ‘suicide inactivation’) during NO-reduction, explaining why no increase is seen at low [NO]. However, we excluded this possibility by plotting the observed maximum rate (at low [NO]) as a function of added amount of enzyme (see S3 Fig). The plot shows a linear dependence for both wildtype and E71^c^D, which is not expected for ‘suicide inactivation’ as each enzyme would have undergone in total fewer turnovers at higher protein concentration. The lower purity of the E71^c^D variant can also not explain the absence of the sigmoidal behaviour as this is already seen in whole cells (over-)expressing *c*NOR. Another possible explanation could be that the inhibitory binding site has been changed in the E71^c^D variant. E71^c^ (E70^c^ in *Ps*. *aeruginosa c*NOR, see Fig 1) is located in a conserved NorC loop, which contains the calcium ligands G71^C^ (backbone C = O) and Y73^C^ (*Ps*. *aeruginosa* numbering). In the crystal structure and MD simulations [5,6], E70^c^ forms hydrogen bonds with the K199 residue from NorB and with various crystallographic/dynamic waters which connect to the Ca^2+^ site (~8Å from the E71^C^ carboxylate oxygens) and the *b*_3_ A-propionate (~6Å from E71^C^) as well as with the *b*_3_ D-propionate (~8Å from E71^C^). Because of these connections, it is possible that the E71^c^D variant alters the heme *b*_3_ midpoint potential, which (if the inhibition site is the oxidised *b*_3_ heme) could alter the inhibition pattern. As indicated by the altered CO-bound spectra in the E71^c^D variant there is a change in the heme *b*_3_ ligation dynamics, indicating a structural change in the heme *b*_*3*_/Fe_B_ active site. However these spectra were also changed in the N322L variant, which retained ~50% of the catalytic activity of wildtype and showed sigmoidal behaviour. Because of the large distance from E71^c^ to Fe_B_ (~10Å), it is not likely that changing the E71^c^ would change the Fe_B_ directly. However, a change in the μ-oxo bridged structure (between the heme *b*_3_ and the Fe_B_) of the oxidised active site could (according to the hypothesis that this is where inhibitory NO binds [21]) affect the inhibition profile. Future studies on this variant are expected to shed more light on the NO inhibition mechanism.

#### Ligand binding

CO rebinding upon flash photolysis is biphasic in *c*NOR. At 1 mM CO, the fast phase has a time constant (τ_1_) of 1304 μs (*k*_on_ ~0.7x10^8^ M^-1^ s^-1^) and the slower phase shows τ_2_~130 μs (*k*_on_ ~7.5x10^6^ M^-1^ s^-1^). The relative contribution to the amplitudes of the slow phase in wildtype is 58% (as described earlier this value depends slightly on preparation, and therefore differs somewhat from the values found in Ref. [7,8,14]). In the H326F, N322L, R121Q, E125D and E71^c^Q/D variants CO binding is substantially slower than in wildtype, indicating that the heme *b*_3_ environment has changed in these variants as also indicated by the altered CO-bound spectra. In the E71^c^Q and N322L variant it is the ratio of the amplitudes of the faster and slower phase of CO binding that has changed rather than the actual rate constants. In the E71^c^D variant only the τ_1_ is larger, while in the R121Q, E125D, N322L and H326F variants both τ_1_ and τ_2_ are increased. We also note that the CO binding rate constants and amplitudes are generally more sensitive to mutagenesis than O_2_ binding (Table 3). O_2_ binding occurs with a τ of 44 μs at 1 mM O_2_, corresponding to a *k*_on_~2.3x10^7^ M^-1^ s^-1^. It’s possible that CO binding dynamics are more sensitive to alterations because they are faster and thus separate subtle conformation differences, that have more time to equilibrate in the case of O_2_ binding. Interestingly O_2_ binding is biphasic in the H326F variant, with a fast phase comparable to wildtype and an additional slow phase (Fig 7).

#### Amplitudes of the ETPT reaction

There is a variation in the amplitudes for the proton-coupled electron transfer (ETPT) in the variants in the continuation of the pathway at 430 nm and 550 nm, but all are smaller than in wildtype (Figs 6 and 7). At 420 nm the ETPT reaction is not even observed for many variants. These observations are probably due to CO rebinding to a part of the population, before electron transfer can take place [7,9]. When the ETPT is completely absent, we can assume that the ETPT reaction has become so slow that CO rebinds to the entire population (the ‘window’ for ETPT to occur is presumably a few seconds, see also [7]). When there is a fractional ETPT reaction, we assume that we have heterogeneity in the *c*NOR population such that one part has very slow ETPT. Such heterogeneity could e.g. be due to partial loss of the calcium ion. Another reason for heterogeneity could be a partly deprotonated proton donor, slowly exchanging (with bulk), such that proton transfer to the proton donor has become slow enough not to occur in the ETPT ‘window’ (slower than in the E58^c^D and K54^c^A variants, where it is still observed). In this case the ETPT could still take place in the *c*NOR fraction that has the donor protonated (as determined by the pH and its p*K*_a_). When the proton exchange between the proton donor and the bulk solvent is so slow, the observed ETPT rate would no longer be pH dependent, but the amplitude will be. This pH dependence of the amplitudes was indeed observed for Y74^c^F/S variants (see S5 Fig), and the ETPT rates of these variants are much less dependent on pH than wildtype (see Fig 8). It should be noted that in the cases where we have plotted the pH dependence of the ETPT (Fig 8), we are plotting the rates in the population with the most ‘wildtype-like’ properties.

### The proton donor in *c*NOR

#### Hypothesis #1 proton donor close to calcium site

The calcium ion in *c*NOR is found in between the A-propionate (7-propionate) of the heme *b*_3_ and the D-propionate (6-propionate) of the heme *b*. Calcium is bound via these two propionates, E135, the carbonyl of G71^c^ (backbone), Y73^c^ and one water molecule [17] (all *Ps*. *aeruginosa* numbering). Calcium thus bridges the two *b* hemes and the NorB and NorC subunit, and therefore plays a structural role. In the qNOR [22] and *cbb*_3_ [23] structures calcium is found in the same location, while in other heme-copper oxidases this location is occupied by two conserved arginine residues [24]. All of the calcium ligands have been shown to be important for *c*NOR activity, the extent and the rate of the ETPT (this article and [9,13]). There was a large p*K*_a_ shift (> 3 pH units) when the calcium ligand E122 (E135 in *Ps*. *aeruginosa c*NOR and Fig 1) was exchanged for an Asp [9]. This led us to propose that the proton donor would be in close proximity [7], but as the mutation surely affects the Ca^2+^-sphere, detailed interpretation is difficult, which is why we here wanted to explore this area further.

The newly constructed and prepared E122 variants gave somewhat different results than earlier reported [9]. E122D gave smaller amplitudes in the ETPT reaction than earlier, while the opposite was observed for E122A [9]. In this study both variants showed an ETPT reaction, but with smaller amplitudes than wildtype. The amplitudes were different in different preparations (and got generally larger when EDTA was left out from the purification for E122D, S4 Fig). These observations lead us to the conclusion that in these variants a varying fraction, but not all, of the enzyme population has lost the bound Ca^2+^ during purification. Thus, the differences in amplitudes of the ETPT could reflect this fractional occupancy of the Ca^2+^ -site.

A tentative fit of the limited pH dependence of ETPT rates for E122A gave a p*K*_a_~8.3 and a *k*_max_~80 s^-1^. Previously data for E122D was fitted with a p*K*_a_>9 (no change in the rate constant was observed for pHs up to 8) and a *k*_max_~160 s^-1^ [9]. Our current preparation of E122D did not show amplitudes large enough to reliably fit any rate constants. Taking current and previous data into account, it is clear that in both E122A and E122D, there is disruption of proton transfer properties such that the p*K*_a_ is significantly higher and the *k*_max_ lower than in wildtype. In Ref. [9] we discussed a model in which the proton donor was interacting with E122, however this was discussed in the context of a *c*NOR model (Ref. [2] from before the structure [5] was known) that placed E122 at the surface, while we now know that E122 is a calcium ligand [5]. However, it is still possible that the proton donor is indeed in close proximity to the E122, see below.

Y74^c^ (Y73^c^ in *Ps*. *aeruginosa*) is also a calcium ligand and is completely conserved in the *c*NOR family [13]. When this residue was exchanged in *T*. *thermophilus c*NOR (Y137^c^F), the produced protein had only 17% of wildtype activity. Also in *P*. *denitrificans*, activity was significantly decreased, but to a smaller extent, upon changing this residue (53% of wildtype in both Y74^C^S and Y74^C^F). Also for the ETPT in single turnover there were significant changes in both Y74 variants (Figs 6 and 8A); the *k*_max_ (maximal rate at low pH) was decreased to 125 s^-1^ (Y74^C^S) and 65 s^-1^ (Y74^C^F) and the p*K*_a_s were both upshifted to p*K*_a_~8.0 (Y74^c^S) and p*K*_a_~7.5 (Y74^c^F). Only limited pH dependence was seen in these variants in the measured pH range. This therefore again indicates that the Ca^2+^ ligation sphere has a large influence on the behaviour of the proton donor, which is probably located close by. The amplitude of the ETPT was larger at low pH than at high pH (S5 Fig), titrating in the same pH range (starting to decrease around pH 8) as the rates do. This could be related to our finding that the amplitudes are also preparation-dependent, which we interpreted as representing a varying degree of heterogeneity (probably related to fractional occupancy of the Ca^2+^-site, see above). In this context the pH dependence of the amplitudes could either be due to: *a)* protons occupying the Ca^2+^-site at low pH, stabilising the protein, or *b)* a larger fractional protonation of the proton donor (which is no longer in rapid equilibrium with bulk pH) at low pH (as also discussed above). Detailed interpretation of the variation in amplitudes is complicated by the experimental procedure for the pH dependence measurements, where the *c*NOR is mixed with the buffer that sets the pH only some 200 ms before the reaction is initiated, leaving not enough time for really slow pH equilibration.

We note that the Ca^2+^-ligand variants (E122A/D and Y74^c^S/F) all show decreased *k*_max_ coupled to upshifted p*K*_a_s of the proton donor with is consistent with Marcus theory for proton transfer. According to this theory (see e.g. [25]) the larger the driving force, Δp*K*_a_ (between the high p*K*_a_ acceptor at the active site and the proton donor), the higher the rate of internal proton transfer (here *k*_max_, see overview in S1 Table).

The E71^c^Q (E70^c^ in *Ps*. *aeruginosa* and Fig 1) variant had no multiple turnover activity, while the E71^c^D variant had 27% NO reduction activity. This indicates that at this position the negative charge is more important than the amino acid structure. The E71^c^D variant had an upshifted p*K*_a_ in the ETPT (from 6.6 to 7.3), but the *k*_max_ (maximum rate at low pH) was similar to wildtype. Taken together we interpret this to mean that the same proton donor is used in this variant as in the wildtype, but that the exchange of the E71^c^ has some effect on this donor. However because of the lower purity and stability of the active site in this variant, we do not want to make detailed conclusions from it.

The R121Q (R134 in *Ps*. *aeruginosa* and Fig 1) variant had only 9% of wildtype multiple turnover activity. The ETPT rate constants at various pH for R121Q could not be fitted reliably and therefore no p*K*_a_ was determined. However, the variant still had a pH dependent ETPT rate and a wildtype-like maximal rate (see Fig 8B). Since the spectra of this variant were also wildtype-like, it seems that it is less affected than many other variants in this region. We already earlier excluded this residue as the proton donor [7] since arginines have been shown to keep their high p*K*_a_ (~12.5) in membrane interiors [26], which doesn’t fit with the observed p*K*_a_ of 6.6. However, the local electrostatics should be changed considerably in this variant, indicating that the proton donor is not in close proximity. Although R121 is the direct neighbour of the Ca^2+^-ligand E122, the side-chain is pointing away from the Ca^2+^ and this conclusion therefore does not exclude a proton donor close to the calcium site.

#### Hypothesis #2 proton donor is D-propionate of heme *b*_3_

Alternatively the proton donor in *c*NOR could have become (or originated from, depending on the rooting of the evolutionary tree; as discussed in for example Ref. [27]) the proton-loading site in the (A, B and C-type) O_2_ reducing HCuOs. This proton-loading site is the site for ‘storing’ the proton that is to be pumped out and has been suggested to be located in the region of the A-propionate of the active site heme (which corresponds in space to the D-propionate of the heme *b*_3_ in *c*NOR) by several groups [28,29]. Therefore we also investigated the possibility that this area forms the proton donor in *c*NOR, by studying two residues with direct interactions to this propionate; H326 and N322 (H339 and N335 in *Ps*. *aeruginosa* and Fig 1). The same propionate-Asn and His interactions are observed in the structure of a C-type HCuO [23] and highly conserved to both B and C-type HCuOs [29].

H326F had no multiple turnover activity and did not show any signal for the ETPT reaction. This severe effect of the H326F variant could be expected if the His itself or the D-propionate of heme *b*_3_ is really the proton donor. However since H326F also showed slow CO binding and seems less stable over time (based on the absorbance spectra), we cannot exclude other reasons for the loss of activity in H326F.

N322L had around 50% NO reduction activity. The measured ETPT rates for N322L between pH 6.5 and 8.5 were very similar to wildtype, but at low pH the rates were substantially lower, and the p*K*_a_ upshifted to ~7.2 (Fig 8B), making the effects qualitatively similar to those in the Ca^2+^-ligands Y74^c^ and E122. As discussed, there were spectral changes in the *b*_3_-CO form over time as well as slowing of CO binding, indicating a change in the heme *b*_3_ conformation. Therefore it is less straightforward to make interpretations about the effects on the proton donor in this variant than in the Y74^c^ and E122 variants, which were more stable.

If we take the information for all the variants in the region of the potential proton donor in *c*NOR into account, our data indicates that the proton donor is found in the heme *b*_3_ propionate region. The strongest effects on proton donation are observed in the variants of the Ca^2+^-ligands E122 and Y74^C^, which via the Ca^2+^ link to the A-propionate. However, changing the D-propionate ligand N322 (to a Leu) shows similar effects, although less severe, on proton transfer.

If we assume one of the propionates is the proton donor, then the increase in p*K*_a_, i.e. destabilisation of the COO^-^ form, could be expected both from a distortion of Ca^2+^-ligation or a loss of a hydrogen-bond from the Asn (N322). Thus, although we cannot at this point say which of the two propionates is the most likely proton donor, our data clearly indicates that the proton donor in *c*NOR is found in the heme *b*_3_ propionate region. As the loading site for pumped protons (PLS) in the proton-pumping HCuOs is presumably located in the same region, it seems likely that there is an evolutionary relationship between them. As we observe qualitatively similar effects on activity and p*K*_a_ of the proton donor when we exchange the Ca^2+^-ligands as well as the N322 (see Fig 1), we believe that there can be quite long-range effects in the propionate area, and that some caution is needed when assigning specific roles to specific amino acids, a caution that extends to studies on other HCuOs. Further mutagenesis studies in this area in *c*NOR, although complicated, is still more straightforward than defining the specific PLS in the proton pumps, and should shed further light on the role of this area in all HCuOs.

## Supporting Information

S1 Fig Multiple turnover rates with NO as a function of pH. Rate of NO consumption (in e^-^ * s^-1^) at different pH values plotted for wildtype (grey triangles) at low [NO]. For comparison the pH dependence of the ETPT reaction rate constants (*k*_obs_ in s^-1^) during O_2_ reduction is also plotted. Experimental conditions for multiple turnover: 50 mM buffer, 50 mM KCl, 0.05% (w/v) DDM, 30 mM glucose, 20 units/ml catalase, 1 unit/ml glucose oxidase, 500 μM TMPD, 20 μM horse heart cytochrome *c*, and 3 mM ascorbate, T = 295K. Experimental conditions for ETPT: ~1–2 μM *c*NOR in 50 mM buffer, 50 mM KCl, and 0.05% (w/v) DDM, [O2] = 1 mM, T = 295K.
(EPS)

S2 Fig **Spectra of *c*NOR wildtype (A) and the studied variants: R121Q (B), E122A (C), E122D (D), E125D (E), N322L (F), H326F (G), E71^c^D (H), E71^c^Q (I) Y74^c^S (J) and Y74^c^F (K)**. Spectra are shown of *c*NOR ascorbate reduced (black line), CO bound (red line), and CO bound after overnight incubation (grey line). Note the break in the y-axis, made in order to emphasize the alpha region.
(EPS)

S3 Fig Multiple turnover rates of the E71^c^D variant are still linearly dependent on protein concentration. Rate of NO consumption (in nM NO * s^-1^) versus *c*NOR concentration for wildtype (circles) and E71^c^D (squares) at low [NO]. Experimental conditions: 50 mM HEPES, pH 7.5, 50 mM KCl, 0.05% (w/v) DDM, 30 mM glucose, 20 units/ml catalase, 1 unit/ml glucose oxidase, 500 μM TMPD, 20 μM horse heart cytochrome *c*, and 3 mM ascorbate, T = 295K.
(EPS)

S4 Fig Reaction between fully reduced *c*NOR wildtype or the E122D variant with oxygen. The traces shown are from wildtype (black), E122D with (cyan) or without (blue) EDTA used during purification. Traces show the absorbance change of *c*NOR in time (with the laser flash at t = 0, which gives an artefact) at 430 nm (A), 420 nm (B) and 550 nm (C, reporting on the heme *c*). At 420 nm and 430 nm the traces were normalized to the CO_off_ step, at 550 nm the traces are normalized as at 420 nm. Experimental conditions: ~1–2 μM *c*NOR in 50 mM HEPES, pH 7.5, 50 mM KCl, and 0.05% (w/v) DDM, [O2] = 1 mM, T = 295K.
(EPS)

S5 Fig The effect of pH on the reaction between fully reduced *c*NOR Y74^c^F and Y74^c^S and O_2_. Fully reduced *c*NOR reacting with oxygen at pH 7.5. Traces show the absorbance change of *c*NOR in time (with the laser flash at t = 0) at 420 nm (A, B) and 430 nm (C, D). For clarity, the laser artefact at t = 0 has been removed. The traces were normalized to the ΔA_CO,off_ step. Experimental conditions: ~1–2 μM *c*NOR in 50 mM HEPES, pH 7.5, 50 mM KCl, and 0.05% (w/v) DDM, [O2] = 1 mM, T = 295K.
(EPS)

S1 Table Properties of *P*. *denitrificans c*NOR variants in proton-coupled electron transfer (ETPT). Data from this study is highlighted in bold, other data is from Ref. [7]. PW = pathway. Conservation and location from Ref. [7].
(DOCX)

## Abbreviations

NOR: NO reductase
*c*NOR: cytochrome *c* dependent NO reductase
PLS: proton loading site
HCuO: heme copper oxidase
ETPT: proton-coupled electron transfer phase
PW: pathway
*P. denitrificans, P. den., Pd.*: *Paracoccus denitrificans*
*Ps. aeruginosa, Ps. aer.*: *Pseudomonas aeruginosa*
*T. thermophilus, T. therm. Tt*: *Thermus thermophilus*
WT: wildtype
