# Correction: Investigating the Proton Donor in the NO Reductase from *Paracoccus denitrificans*

**DOI:** 10.1371/journal.pone.0155365

**Published:** 2016-05-20

**Authors:** The PLOS ONE Staff

There is an error in the second sentence of the “Ligand Binding” subsection within the Discussion. The correct sentence is: At 1 mM CO, the fast phase has a time constant (τ_1_) of 14 μs (*k*_on_ ~0.7x10^8^ M^-1^ s^-1^) and the slower phase shows τ_2_~130 μs (*k*_on_ ~7.5x10^6^ M^-1^ s^-1^). The publisher apologizes for this error.

There is an error in the caption for Fig 3. The number ‘108’ should be ‘10^8^’. Please see the complete, correct Fig 3 caption here. The publisher apologizes for this error.

**Fig 3. pH dependence of the proton-coupled electron transfer (ETPT) in the reaction between O_2_ and fully reduced cNOR variants at the entrance of proton transfer pathway 1.**

The rate constants of the ETPT plotted as a function of pH: wild type (black circles), K54^c^A (red squares), E58^c^Q (blue triangles down), E58^c^D (green triangles up). The data from E58^c^Q, K54^c^A and wildtype are from Ref. [7,8] (added for comparison). The data for wildtype was fitted to a pKa ~6.6 and a *k*_max_ (maximal rate at low pH) of ~250 s^-1^ (black line). The data for E58^c^Q was fitted to a p*K*a of ~5.8 and a *k*_max_ of ~50 s^-1^ (blue line, data and fit from from Ref. [7]). The data for K54^c^A was fitted to a p*K*a of ~6.4 and a *k*_max_ of ~250 s^-1^ (red line, data and fit from Ref. [7]) and for E58^c^D to a p*K*a of ~6.4 and a *k*_max_ of ~250 s^-1^ (green line). Note that data points for K54^c^A as well as the E58^c^D do not follow the fit around pH 7–7.5, and also plotted (see text) are two theoretical diffusion rate constants (*k*_diff_*[H^+^]) as a function of pH (= -log[H^+^]), assuming a *k*_diff_ of 2.5*10^8^ M^-1^ s^-1^ in K54^c^A (red dashed line) and 3.5*10^8^ M^-1^ s^-1^ in E58^c^D(green dashed line).

There is an error in the Abstract. The first paragraph should be included in the Abbreviations. The Abstract should begin with the first sentence of the second paragraph: Bacterial NO reductases (NORs) are integral membrane proteins from the heme-copper oxidase superfamily. Most heme-copper oxidases are proton-pumping enzymes that reduce O_2_ as the last step in the respiratory chain.
