# Supplementary material for: Investigating the Proton Donor in the NO Reductase from Paracoccus denitrificans
Source: PLoS One. 2016 Mar 31;11(3):e0152745. doi: 10.1371/journal.pone.0152745 (PMC4816578; doi:10.1371/journal.pone.0152745)
Supplement: S1 Table — Data from this study is highlighted in bold, other data is from Ref. [7]. PW = pathway. Conservation and location from Ref. [7]. (DOCX) [file pone.0152745.s006.docx]

| **Res. in *P. aer.* (conservation) Location** | **Mutation in**  ***P. den.*** | **p*K*_a_** | ***k*_max_ (s^-1^)** |
| --- | --- | --- | --- |
|  | WT | 6.61 ± 0.05 | 250 ± 7 |
| E57^C^ (77% E, rest Q, H, D)  Entrance proton PW 1 | E58^C^Q | 5.8 ± 0.1 | 49 ± 2 |
| E57^C^ (77% E, rest Q, H, D)  Entrance proton PW 1 | **E58^C^D** | **6.4 ± 0.1**^a)^ | **250 ± 20** |
| K53^C^ (100% K)  Entrance proton PW 1 | K54^C^A | 6.4 ± 0.1^a)^ | 250 ± 10 |
| E77^c^ (76% E, 24% D)  In conserved loop with Ca^2+^ ligands G71^C^ and Y73^C^, forms many hydrogen bonds. | **E78^c^D** | **6.6 ± 0.1** | **240 ± 10** |
| E70 ^C^ (80% E, rest N, H, Q, S, F)  In conserved loop with Ca^2+^ ligands | **E71^C^D** | **7.3 ± 0.1** | **260 ± 20** |
| Y73^C^ (99% Y, rest R)  Ca^2+^ ligand | **Y74^C^S** | **8.0 ± 0.4** | **125 ± 30** |
| Y73^C^ (99% Y, rest R)  Ca^2+^ ligand | **Y74^C^F** | **7.5 ± 0.2** | **65 ± 7** |
| E135 (88% E, 9% K, rest: H, S, V, P)  Ca^2+^ ligand | **E122A** | **~ 8.3** | **~ 80** |
| N335 (100% N)  H-bond D-prop. heme *b*_3_ | **N322L** | **7.2 ± 0.1** | **138 ± 7** |

a) The simple one-p*K*a fit does not fit the data points at pH>7.
